# From Magnetic Moment to Magnetic Particle Imaging: A Comprehensive Review on MPI Technology, Tracer Design and Biological Applications

**DOI:** 10.3390/pharmaceutics18040497

**Published:** 2026-04-17

**Authors:** Alessandro Negri, Andre Bongers

**Affiliations:** Biological Resources Imaging Laboratory, Mark Wainwright Analytical Centre, University of New South Wales (UNSW), Sydney, NSW 2052, Australia

**Keywords:** magnetic particle imaging (MPI), superparamagnetic iron oxide nanoparticles (SPIONs), nanomedicine, magnetic nanoparticle, tracer design, quantitative tomographic imaging, cell tracking, theranostics

## Abstract

**Background/Objectives**: Magnetic nanoparticles have emerged as powerful tools for biomedical imaging, targeted drug delivery, and hyperthermia therapy. Magnetic particle imaging (MPI) is among the most promising technologies built around its properties: a radiation-free, quantitative tomographic modality that detects superparamagnetic iron oxide nanoparticles (SPIONs) directly against a biologically silent background. This review synthesizes MPI’s physical principles, nanoparticle design strategies, and preclinical applications within the broader landscape of magnetic material engineering for biomedical use. **Methods**: A systematic review was conducted covering MPI signal generation and image reconstruction, nanoparticle core synthesis and surface coating approaches, and preclinical applications, spanning cell tracking, oncological imaging, vascular perfusion, neuroimaging, and MPI-guided theranostics. Studies were selected to provide quantitative benchmarks and direct comparisons with competing modalities where available. **Results**: MPI delivers signal-to-background ratios above 1000:1, iron-mass linearity at R^2^ ≥ 0.99, regardless of tissue depth, and acquisition rates up to 46 volumes per second. Tracer architecture—encompassing single-core particles, multicore nanoflowers, and stimuli-responsive cluster designs—is the primary determinant of sensitivity, environmental robustness, and theranostic capability. Preclinical results include detection of cell populations in the low thousands, earlier ischaemia identification than diffusion-weighted MRI, real-time drug release quantification, and spatially confined tumour hyperthermia. Three translational bottlenecks are identified: the absence of a clinically approved tracer with optimal relaxation dynamics, hardware performance losses when scaling to human-bore systems, and overestimation of passive tumour accumulation in murine models. **Conclusions**: MPI illustrates how progress in magnetic material design directly expands clinical imaging and theranostic possibilities. Successful translation will require indication-driven, interdisciplinary development that integrates materials science, scanner engineering, and regulatory strategy in parallel.

## 1. Introduction

Modern medicine is based on rapid and accurate imaging to guide both diagnosis and therapeutic intervention. Over the past few decades, continuous refinements in magnetic resonance imaging (MRI) [1,2,3], computed tomography (CT) [4,5], positron emission tomography (PET) [6,7], and ultrasound (US) [8,9] have vastly expanded the accessible spectrum of anatomical, functional and molecular information. Advances in molecular tracers and contrast agents have further broadened the biological space that can be probed [10], while hybrid systems, such as PET/CT, have demonstrated how multimodal integration can improve both sensitivity and anatomical precision [11].

Despite these achievements, a significant capability gap remains currently; no single modality can simultaneously provide radiation-free, strictly quantitative, deep-tissue imaging, and there is always a compromise to make. PET and CT examinations rely on ionizing radiation, which restricts their use in repeated or longitudinal imaging [12]. MRI and CT offer structural clarity, but their contrast agents carry dose-dependent safety concerns that limit high-frequency scanning or high-dose administration [13,14,15]. These constraints become particularly restrictive when the clinical question demands serial and/or quantitative imaging [16], for example, tracking therapeutic cell populations [17], detecting micro-metastatic disease [18], assessing dynamic perfusion in acute care [19], or monitoring nanoparticle-based interventions [15]. In such contexts, cumulative radiation burden or contrast agent toxicity can often act as hard ceilings on study design, reducing patient eligibility and narrowing the scope of research [20].

This unmet need defines the operational space for magnetic particle imaging (MPI). MPI is a rapidly emerging tomographic technique that is uniquely positioned to bridge the gap by offering a quantitative, radiation-free platform that retains high sensitivity and deep-tissue performance. Its tracer agents, superparamagnetic iron oxide nanoparticles (SPIONs), are iron oxide cores that produce a strong, nonlinear magnetization response under an oscillating magnetic field while retaining zero net magnetization at rest. Because background tissue generates no such signal, MPI allows for straightforward and absolute quantification of iron (Fe) content. This makes it an imaging platform ideally suited for high-frequency, longitudinal studies in modern medicine, in both preclinical and translational settings. From a clinical perspective, MPI is best viewed not as a direct competitor to MRI, CT, or PET, but more as a complementary tool. It offers solutions for specific, high-impact problems where radiation, tracer toxicity, or nonlinear kinetics already limit study design. While MPI hardware and tracers remain in a late preclinical to early translational phase, framing the modality as a targeted, high-impact solution is a more realistic and promising approach than expecting it to replace existing modalities across the board. In this review, we aim to provide an integrated overview of MPI, from physics to biomedical applications. Specifically, we summarize the core principles of signal generation and image reconstruction, examine how nanoparticle core architecture and surface design govern imaging performance, review major synthesis and coating strategies, and assess the strengths, limitations, and translational barriers of current preclinical MPI applications.

## 2. Magnetic Particle Imaging: Principles, Strengths, and Constraints

At its core, MPI represents a fundamental difference from traditional imaging methods, such as X-ray or MRI. Rather than generating signals from endogenous tissue, MPI directly measures the nonlinear magnetization dynamics of SPIONs in response to an alternating magnetic field (AMF) [21]. Because biological tissues are transparent to electronic magnetization, the resulting signal originates only from the tracer, yielding background-free, radiation-free images, with deep-tissue sensitivity and negligible signal attenuation, often exceeding signal-to-background ratios of 1000:1 [11]. This tracer exclusivity, combined with an inherent linear relationship between SPION mass and signal intensity, provides the physical basis for absolute quantification without tissue-depth attenuation correction [22]. In practice, however, achieving this quantitative accuracy requires standardized system calibration to account for tracer-specific relaxation behaviour and environmental factors. Critically, this shifts the optimization paradigm away from “maximizing contrast agent effect” towards “maximizing tracer magnetic fidelity”, transforming the nanoparticle from a passive contrast agent into the active driver of imaging performance [11,23,24].

However, as in PET, MPI is subject to partial volume effects (PVEs) arising from the system’s finite spatial resolution. When tracer-bearing structures are similar in size to, or smaller than, the effective width of the system’s point spread function (PSF), the reconstructed image fails to accurately reflect the true local tracer distribution, a phenomenon known as “spill-over”. Formally, the reconstructed tracer map can be described as the true nanoparticle concentration distribution convolved with the system’s PSF, such that small structures appear broadened and their peak signal is reduced [25]. This bias is particularly consequential when quantifying small amounts of Fe confined to narrow compartments, for instance, sparse labelled-cell deposits, which may appear artificially dim, or vascular stenoses, where sub-resolution luminal narrowing is smoothed out and consequently underestimated [25]. To address these limitations, quantitative MPI pipelines typically incorporate recovery coefficients (phantom-derived or size-dependent) to compensate for resolution-related signal loss, alongside deconvolution filters (e.g., Wiener-type methods), designed to reverse PSF-induced blurring while limiting noise amplification, ultimately improving quantitative accuracy in sub-resolution volumes [25,26]. Notably, some preclinical systems have demonstrated acquisition rates of 46 volumes/second in complex biological environments, making MPI fast enough for high-speed dynamic studies [27,28].

Where tracer properties and imaging conditions are well matched, MPI’s signal-to-mass linearity offers a principled basis for quantitative assessment, a feature that has been exploited in applications such as vascular and perfusion imaging [29,30], neurotrauma assessment [31], and tracking SPION-labelled therapeutic cells in regenerative medicine and immunotherapy [18,29,32,33,34,35]. The degree of quantitative accuracy achievable in practice, however, varies across these scenarios and depends on tracer characterization, system spatial resolution relative to the structure of interest, and the magnitude of relaxation and reconstruction-related artefacts, as outlined in the preceding section. Beyond tracking, MPI provides a quantitative framework for nanoparticle-enabled theranostics [36]. Because SPIONs can be engineered with tailored physicochemical and magnetic properties, MPI integrates naturally with magnetic fluid hyperthermia [33,37], magnetically guided drug delivery and release [38,39,40], targeted nanoparticle accumulation [41], and rapid nano-rewarming of cryopreserved organs [42], and emerging magneto-mechanical actuation strategies, in which scanner fields are used to apply forces or torques to magnetic carriers, enabling steering alongside imaging [43]. Closely related magnetoelectric sensor approaches have likewise demonstrated higher-harmonic-based localization of magnetic nanoparticle distributions and magnetically labelled cells, suggesting a complementary route toward magnetic particle mapping [44]. These developments further reinforce the role of MPI as a simultaneous platform for planning, guiding, and verifying therapeutic interventions in real-time. By enabling the precise measurement of biodistribution, bioaccumulation, and clearance, MPI can also accelerate the design and iterative validation of next-generation nanomedicines.

Yet the very trait that defines MPI’s strength also imposes its primary limitation. Without hybrid imaging (e.g., MPI–CT/MRI) or external a priori structural data, MPI cannot distinguish tissues or discriminate between compartments with comparable SPION distributions. Particle accumulation cannot be assigned to viable or necrotic regions, nor can it distinguish between anatomically different tissues with similar tracer content. Additionally, while the linearity of the signal-to-mass relationship simplifies quantification, it does not entirely remove artefacts arising from relaxation effects, trajectory imperfections, or undersampling; it only makes them easier to model [15,45].

In our view, realistic clinical deployments will therefore pair MPI closely with anatomical modalities (MRI, CT, and US) and use its strengths primarily for quantitative tasks, such as cell counting, perfusion mapping, and therapy monitoring, rather than for standalone diagnostics.

## 3. From Signal to Image: Field-Free Region Encoding and Reconstruction Strategies

MPI encodes spatial information by recording the nonlinear magnetization signature of SPIONs when subjected to controlled magnetic field configurations. While the specific hardware implementation varies between manufacturers, a typical 3D MPI scanner is built around three core hardware units: the selection- and focus-field coil unit, the drive-field coil unit, and the receive coil unit. The selection field is a static, strongly inhomogeneous gradient field which cancels the net field at its centre while saturating the particles everywhere else in the Field of View (FOV). This creates the field-free region (FFR), the only spatial location where SPIONs remain magnetically unsaturated and capable of generating a detectable signal. Depending on the scanner architecture, this FFR is shaped as either a field-free point (FFP) or a field-free line (FFL) [16,34,46].

**Figure 1:**
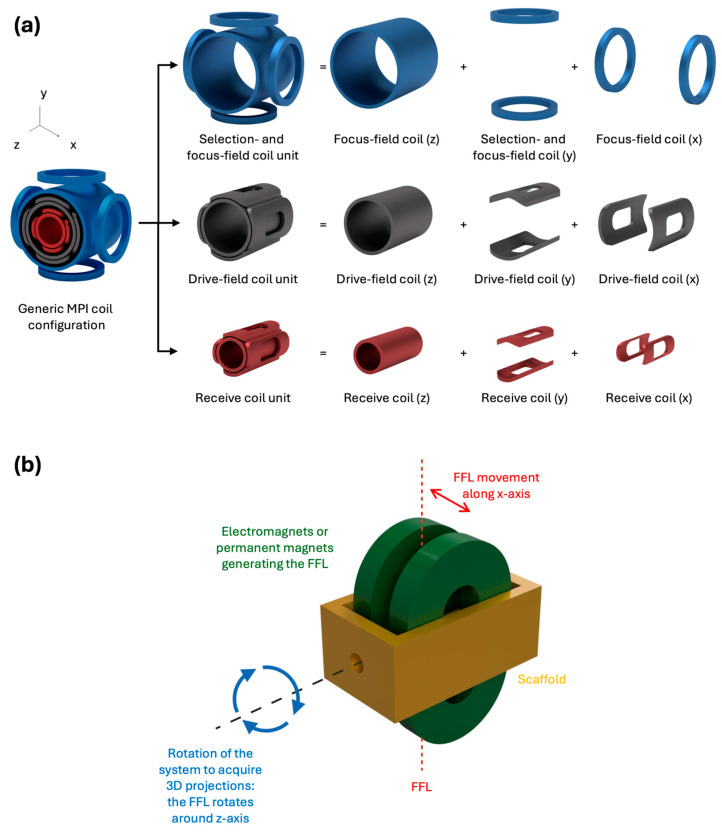
A pictorial representation of two representative MPI coil configurations. (**a**) A generic FFP-based MPI scanner. In blue, the selection- and focus-field coil units. A Maxwell coil pair in the y-direction generates the selection field, featuring an FFR at the centre. By superimposing currents in the same coils, the FFR can be moved in the y-direction, using the focus field. A Helmholtz coil pair directed in the x-direction generates a second focus field for moving the FFR in the x-direction. In the z-direction, a solenoidal focus-field coil is used. In gray, the drive-field coil unit, consisting of three orthogonal components. While the drive fields in the x- and y-directions are realized by saddle-shaped Helmholtz coils, the z-drive-field coil is of cylindrical shape and positioned inside the x- and y-drive-field coils. In red, the receive coil unit, consisting of three orthogonal components. While the receive coils in the x- and y-directions are realized by saddle-shaped Helmholtz coils, the z-receive coil is of cylindrical shape and positioned inside the x- and y-receive coils. (**b**) A representative FFL-based MPI scanner. Two electromagnets or permanent magnets (green) mounted on a structural scaffold (yellow) generate a 1D FFL oriented perpendicular to the *z*-axis. To acquire 3D images, the entire assembly rotates around the *z*-axis, collecting projection data at multiple angles in a gantry-like fashion.

In FFP-based systems, the focus field complements the selection field by extending the effective imaging volume. Generated by Helmholtz coil pairs along the x-direction, and a cylindrical coil along the *z*-axis, it applies low-frequency shifts that move the FFR along the x- and z-directions. The drive-field coil unit consists of three orthogonal coil sets, typically saddle-shaped Helmholtz pairs for the transverse axes and a cylindrical shape for the longitudinal axis. It generates high-frequency oscillating homogeneous fields (typically an AMF) in the 1–100 kHz range, steering the FFR along three-dimensional trajectories [47]. The receive coil unit detects the weak voltages induced by the rapidly changing particle magnetization within the FFR via Faraday induction. Arranged in an analogous three-axis configuration to the drive unit, it captures the full three-dimensional magnetization vector. In some designs, the drive and receive coils are co-located to bring the sensors as close to the subject as possible. Figure 1a offers a pictorial representation of a generic MPI system.

FFL-based systems replace the FFP with a 1D line-shaped one, achieved through the superposition of two orthogonal Maxwell coil pairs or through specific permanent magnet arrangements (Figure 1b). Because the zero-field region extends along an entire line, approximately 10 times more SPIONs can contribute to the signal simultaneously, yielding substantially higher sensitivity and Signal-to-Noise Ratio (SNR) compared to FFP systems. However, to encode a full 3D image, the FFL must be both translated and rotated by at least 180° across the FOV. This requirement naturally calls for a gantry-based mechanical design, as adopted in commercial systems, such as the Magnetic Insight MOMENTUM scanner, where the coil assembly rotates around the subject. The primary trade-offs relative to FFP systems are greater hardware complexity, higher power demands, and increased sensitivity to gradient inhomogeneities that can introduce reconstruction artefacts. Figure 1b illustrates a representative FFL coil configuration.

**Figure 2:**
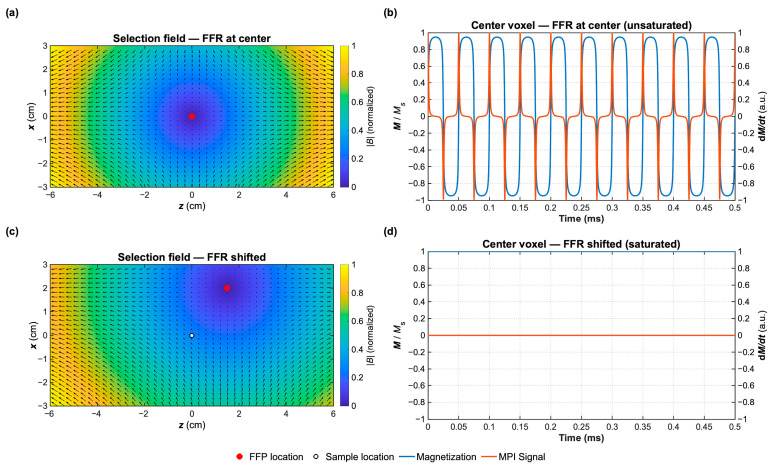
The principle of signal generation and spatial encoding in magnetic particle imaging (MPI). (**a**) shows a schematic of the MPI FOV with the FFR positioned at the centre, where SPIONs are unsaturated and responsive to the drive field. (**b**) shows the time-domain traces of the applied drive field and the resulting nonlinear SPION magnetization response *dM*/*dt* (orange). (**c**) shows the FFR shifted away from the sample position, so SPIONs at the sample (still at the centre of the FOV) experience a strong selection field and become magnetically saturated. (**d**) shows the corresponding receive signal, demonstrating no detectable contribution from the saturated particles, thereby illustrating MPI’s spatial selectivity and background-free contrast.

However, regardless of the system’s architecture, only SPIONs located within the FFR can follow the rapid magnetization oscillations induced by the drive-field coils, generating a voltage in the receive coil, rich in higher-order harmonics that is the direct hallmark of their nonlinear magnetization dynamics [15,18,45]. In contrast, particles located outside of the FFR experience strong fields that saturate their magnetic moments, making them effectively silent and not contributing to the received signal. Figure 2 illustrates the resulting spatial selectivity: Figure 2a,b show the FFR positioned at the FOV centre, where SPIONs are unsaturated, and their nonlinear response *dM/dt* is clearly resolved. Figure 2c,d show the FFR moved away from the sample, causing full magnetic saturation and a complete absence of detectable signal.

As the scanner sweeps the FFR across the FOV, mechanically or electronically, the system records a time series whose instantaneous voltage directly corresponds to the local tracer concentration at the FFR location. This one-to-one mapping between FFR position and signal amplitude forms the basis of MPI’s spatial encoding [13,17,26,48,49]. To convert the raw encoded voltage signal into a high-fidelity image, the field relies on two main reconstruction approaches: the system matrix (SM) method and the X-space method, which together span a continuum of modelling assumptions.

### 3.1. Frequency-Domain Reconstruction: System Matrix Calibration and Inversion

Since its introduction by Gleich and Weizenecker in 2005 [21], alongside the development of magnetic particle imaging (MPI), the system matrix (SM) method has remained the benchmark for quantitative tomographic reconstruction. Conceptually, the SM approach models the “scanner–tracer–environment” triad as a complex transfer function, Ŝ, which is empirically measured rather than analytically approximated, representing, in effect, the “physical fingerprint” of the imaging system. It is acquired during a calibration phase by recording the multi-harmonic frequency response of a point-like “delta sample” (a small tracer volume) at each voxel throughout the FOV [21,50,51].

Mathematically, image formation is defined by the linear system:

(1) $$\hat{S}c=\hat{u}$$

where $\hat{S}$ is the SM containing all the frequency components of the calibration scans, *c* represents the unknown spatial distribution of tracer concentration, and $\hat{u}$ is the Fourier-transformed multi-harmonic voltage signal [21,52].

The primary strength of this framework is its realism: because the matrix is measured directly, it inherently accounts for hardware imperfections, complex field geometries, and the nonlinear behaviour of particles [19,53], factors that are often oversimplified in other analytical reconstruction models [19]. However, this precision comes at the logistical cost of extensive calibration that scales linearly with both FOV size and desired spatial resolution. This method is also highly specific: an SM acquired with a specific tracer formulation or in a specific environment (e.g., water) may not accurately reconstruct data acquired with other particles or biological media (e.g., blood), where viscosity can be different and particle mobility altered [52,54,55,56].

Reconstruction requires inverting the system matrix, but the MPI SM is inherently ill-conditioned, meaning that direct inversion leads to severe noise amplification. Here, we report only two of the basic reconstruction approaches, but to better understand the reconstruction approaches, please refer to [57,58,59,60]. Singular Value Decomposition (SVD) offers the most mathematically rigorous solution, decomposing the SM into fundamental spatial and frequency components to yield an exact inversion. However, its computational demands scale nonlinearly with matrix dimensions, rendering it prohibitive for high-resolution 3D imaging where system matrices can exceed terabytes [61], and its extreme sensitivity to noise in high-frequency components requires aggressive truncation that can paradoxically degrade spatial resolution, despite the mathematical exactness of the approach. Tikhonov regularization addresses the same ill-conditioning through a different strategy: rather than decomposing the matrix, it reformulates the optimization problem to include a penalty term that bounds the solution norm [62]. This makes it computationally tractable and robust across diverse imaging conditions, establishing it as the workhorse for routine preclinical imaging.

### 3.2. Time-Domain Reconstruction: X-Space Approach

In 2010, Goodwill and Conolly introduced the X-space reconstruction approach, a paradigm that operates in the time domain, instead of frequency, to provide real-time tomographic mapping [63]. Unlike the empirically driven SM method, X-space treats the MPI scanner as an approximately Linear and Shift-Invariant (LSI) system [63,64]. Linearity means that the total measured signal equals the linear sum of signals generated by multiple tracer sources, whereas shift invariance implies that the system’s response depends only on the relative position between the tracer and the FFR, and not on the absolute spatial location of the tracer within the FOV.

Under this framework, image formation proceeds through a computationally efficient two-step process: first, a velocity compensation step normalizes the induced voltage by the instantaneous FFR speed to account for the signal’s dependence on the rate of spatial traversal, after which the compensated signal is gridded into the spatial domain according to the FFR trajectory [28]. By avoiding high-dimensional matrix inversion entirely, X-space enables fast image reconstruction with minimal latency, a critical advantage for dynamic applications. The resulting ‘native’ MPI image represents the spatial convolution of the tracer distribution with the system’s PSF, mathematically defined as the derivative of the Langevin function [63].

The X-space framework rests on several simplifying assumptions that introduce known failure modes. The adiabatic assumption requires that nanoparticle magnetic moments align instantaneously with the AMF and that the FFR maintains a unique, well-defined position at all times [64]. In practice, this breaks down as particle diameters approach the ‘Relaxation Wall’, where magnetoviscous drag induces a phase lag between the AMF and the particle response, manifesting as spatial blurring and signal suppression [61,65]. A separate practical challenge arises from the direct feedthrough contamination: the excitation coil induces a signal 10^9^ to 10^10^ times stronger than the tracer response, requiring high-pass filtering of the fundamental frequency (*f*_0_) [16]. This removes the direct current (DC) component of the image, and disrupts the strict LSI hypothesis; however, the removed baseline information is recoverable by acquiring multiple overlapping partial fields of view (pFOVs) and sequentially minimizing the error between adjacent signals [26,64].

In practice, the choice between SM and X-space reconstruction reflects a fundamental trade-off between physical realism and computational efficiency and is ultimately governed by the demands of the application. The SM approach is preferred when absolute quantification is paramount, and the imaging environment is stable enough to justify the calibration overhead. X-space, by contrast, excels where speed and adaptability matter most: its ‘calibration-light’ nature and real-time throughput make it the superior choice for high-speed dynamic studies like angiography or interventional guidance [66,67]. Hybrid strategies that combine the physical fidelity of SM calibration with the computational efficiency of X-space gridding represent an active area of development, and the optimal reconstruction pipeline will increasingly depend on the specific clinical or preclinical task at hand.

## 4. Magnetic Nanoparticle Tracers: Physical Principles and Design Architecture

### 4.1. Tracer Magnetization and the Point Spread Function: The Langevin Framework

As established in Section 2, the sensitivity, spatial resolution, and detection limits of MPI are determined primarily by the physical properties of the tracer, with scanner hardware only playing a secondary role [34]. Understanding why requires examination of how the tracer’s magnetization dynamics translate directly into imaging performance.

**Figure 3:**
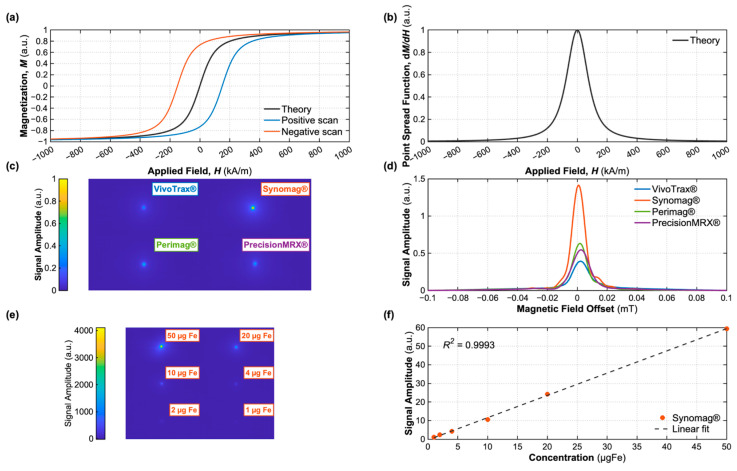
(**a**) The idealized Langevin magnetization curve M(H) and the representative positive/negative field sweeps. (**b**) The normalized derivative $\frac{dM}{dH},$ representing the intrinsic tracer point spread function (PSF). (**c**) Representative reconstructed MPI images (commercial nanoparticles phantom examples). (**d**) Example PSF profiles comparing commercial tracers. (**e**) MPI images of the Synomag phantom dilution series (50, 20, 10, 4, 2, and 1 µgFe in 50 µL), demonstrating sensitivity across a wide dynamic range. (**f**) The corresponding MPI signal as a function of the iron concentration, showing a linear relationship (R^2^ = 0.99), confirming the intrinsic quantitative nature of MPI.

In the imaging process, tracer particles are repeatedly driven from positive to negative magnetization to generate an induction signal in the receive coils. To gain a fundamental understanding of this process, it is commonly assumed that the magnetic particles follow the applied field instantaneously and they behave adiabatically [64]. In this so-called “Langevin model” [18], the particle magnetic moment m(H) can be written as:

(2) $$m\left(H\right)=\mu L\left(\alpha \right)\;\mathrm{where}:\;L\left(\alpha \right)=\coth(\alpha )\;-\;\frac{1}{\alpha };\;\mu =M_{s}\frac{\pi d^{3}}{6};\;and\;\alpha =\;\frac{\pi M_{s}d^{3}}{6k_{b}T}\mu_{0}H$$

where *µ* is the particle saturation magnetic moment, *M_s_* is the saturation magnetization of the core material, *d* is the magnetic core diameter, *µ*_0_ is the vacuum permeability, *H* is the applied magnetic field strength, *k_B_* is the Boltzmann constant, and *T* is the absolute temperature. It should be noted that the Langevin function is strictly valid only for magnetic nanoparticles lacking magnetocrystalline anisotropy; nevertheless, it is widely employed as a practical approximation for particles with finite anisotropy, as it captures the essential features of the magnetization response [68]. Figure 3 summarizes the tracer response predicted by the Langevin model. Figure 3a shows the theoretical magnetization curve M(H) with positive and negative scans, and Figure 3b its derivative. Figure 3c shows MPI images of four different commercial SPIONs, and Figure 3d shows the PSFs of the same particles.

The resulting induction signal is proportional to the derivative of this function with respect to the field variation *dM/dH* [11]. The typical sigmoidal magnetization curve, therefore, yields a “bell-shaped” PSF, whose width and peak height are determined by the steepness of the central slope of the magnetization curve, defining the effective spatial resolution function of the system. Specific to MPI, this PSF is largely dictated by the tracer itself rather than the scanner components, which is why the tracers are often described as having their own intrinsic PSF. Quantitatively, relative signal strength and resolution of a specific tracer are reported by measuring peak height and width of the PSF (typically Full Width at Half Maximum, FWHM) of its PSF [69]. For a standard FFP scanner, spatial resolution (*x*) can be estimated from the PSF FWHM as:

(3) $$x\approx \frac{{PSF}_{FWHM}}{G},$$

where the FWHM of *dM/dH* and static selection gradient (*G*) are the governing parameters [53,63,70]. A key advantage of MPI is that the detected signal is also directly proportional to the local tracer concentration, enabling absolute quantification of iron mass. As illustrated in Figure 3e,f, this linearity holds across a wide dynamic range: serial dilutions of Synomag^®^ (50, 20, 10, 4, 2, and 1 µgFe in 50 µL) yield a signal vs. iron concentration relationship of R^2^ = 0.99, confirming that the MPI signal can serve as a direct, quantitative readout of the tracer amount without the saturation effects that complicate modalities such as MRI.

Within this adiabatic Langevin framework, tracer PSF performance is critically determined by two particle parameters. First, the particle core diameter (*d*): the PSF width sharpens, and the spatial resolution improves approximately with the cube of the magnetic core diameter, as larger magnetic cores exhibit steeper magnetization curves and greater magnetic moment, with sensitivity scaling proportionally with the core volume [71,72]. Second, the saturation magnetization (M_s_): a higher M_s_ increases signal amplitude and improves the spatial confinement of the PSF. A steeper M(H) curve allows for more localized signal generation, and low magnetic anisotropy is desirable, as it enables faster reorientation of the magnetic moments under the AMF, improving responsiveness [11]. From this (simplified) Langevin model, the optimization strategy appears straightforward: it synthesizes larger iron oxide cores with the highest possible saturation magnetization.

### 4.2. Beyond the Adiabatic Ideal: Brownian and Néel Relaxation in MPI Tracers

In real systems, nanoparticles (NPs) cannot align instantaneously with the fast, high-amplitude drive fields of the MPI scanner. Instead, they exhibit a finite temporal lag relative to the applied field, magnetic relaxation, leading to reduced signal amplitude, spatial blurring, and apparent shifts in the tracer location [73]. Two distinct physical mechanisms contribute to total relaxation:

1. Brownian relaxation describes the rotation of the entire particle within the surrounding medium. The Brownian relaxation time (τ_B_) is sensitive to the hydrodynamic volume (V_H_) of the nanoparticle (core plus coating), the viscosity (η) and temperature (T) of the surrounding medium [74]. τ_B_ is described as:
  (4) $$\tau_{B}=\frac{3\eta V_{H}}{k_{B}T},$$
  where *k*_B_, *T*, η, and *V*_H_ are the Boltzmann constant, temperature, viscosity of the solvent, and hydrodynamic volume of the SPIONs, respectively.
2. Néel relaxation describes the internal rotation or flipping of the particle’s magnetic moment relative to its crystal lattice, while the physical particle remains stationary. The Néel relaxation time (τ_N_) depends primarily on the particle’s magnetic anisotropy (K) and core volume (V_C_), with an exponential dependence on the cube of the core diameter [75]. τ_N_ is described as
  (5) $$t_{N}=t_{0}\;exp\left(\frac{KV_{C}}{k_{B}T}\right)$$
  where t_0_ is the attempt time characteristic of the material (time between successive attempts by a magnetic nanoparticle’s moment to flip over its internal energy barrier due to thermal fluctuations), *K* is the magnetic anisotropy energy density, *V_c_* is the effective magnetic core volume, and *k*_B_ and T are the Boltzmann constant and temperature, respectively. The distinction between the core volume (V_C_) and hydrodynamic volume (V_H_) is critical; Néel relaxation depends only on the magnetic core volume, while Brownian relaxation scales with the larger hydrodynamic volume, which includes the particle coatings as well.

### 4.3. The Physical Limits of Single-Core Tracers: The Relaxation Wall

The Langevin assumptions that increasing core size improves performance indefinitely break down through relaxation effects [11]. A fundamental physical ceiling, commonly referred to as the “relaxation wall”, arises from the finite timescale of Néel relaxation. For single-core magnetite particles driven by standard MPI excitation fields (typically 10–100 kHz), this limit occurs at approximately 25 nm [76,77]. As the magnetic core volume increases, τ_N_ approaches the drive-field period, and the particle’s magnetic moment can no longer track the applied field instantaneously, lagging behind by a finite phase angle that fundamentally deviates from the equilibrium Langevin assumption [69]. In the reconstructed image, this manifests as PSF broadening and characteristic asymmetry that worsens steeply as the core exceeds this “magnetic diameter threshold”. Because τ_N_ grows exponentially with V_c_, this signal degradation becomes severe within a narrow size window above the critical core diameter, creating a “sensitivity–resolution paradox” for single-core NPs: the conditions that maximize raw signal intensity are exactly those that degrade spatial precision [78,79].

For particles larger than the critical Néel diameter, Brownian relaxation may become the dominating relaxation mechanism [77,80]. Because τ_B_ scales linearly with hydrodynamic volume and medium viscosity, it introduces a profound environmental dependency: hydrodynamic shell thickness, particle aggregation, and viscosity all vary considerably across biological compartments [81]. A tracer engineered for fast Brownian rotation in a dilute aqueous suspension will behave very differently in viscous biological media, such as blood, synovial fluid, or intracellular endosomal compartments [82,83]. This gives rise to the “Mobility Paradox”: a tracer optimized for Brownian-dominated relaxation in a calibration phantom may exhibit dramatically degraded performance in vivo, particularly upon cellular endocytosis, where Brownian rotation is blocked entirely, and the particle is forced to rely on Néel switching alone. If the core diameter exceeds ~25 nm, the resulting τ_N_ can be exponentially long, leading to signal losses [69].

## 5. Magnetic Nanoparticle Tracer Design: From Physical Principles to Engineered Multi-Component Systems

The theoretical framework established in the previous section defines the physical space within which MPI tracer performance is determined. Bridging this to real-world imaging requires recognizing that a magnetic tracer is an engineered multi-component system, whose performance emerges from the interplay of three interdependent elements: (i) the magnetic core, the primary signal generation unit; (ii) the surface coating, which governs colloidal stability, hydrodynamic size, and biocompatibility; and (iii) optional targeting moieties, which direct the tracer to specific biological sites [11,47]. Each component influences the others in complex and often counterintuitive ways, collectively defining imaging performance, pharmacokinetics, and safety profiles [47]. This section focuses on the physical–magnetic properties of the core and on the distinct design logic of single-core vs. multicore particle architectures.

### 5.1. The Magnetic Core: Key Parameters Governing the Tracer Signal

Building on the Langevin framework established in Section 4.1, both the spatial resolution and sensitivity scale with the cube of the core diameter, and higher M_s_ lowers detection limits by increasing signal output per unit Fe mass. In practice, however, this optimization pathway is constrained by a suite of physical and structural properties that collectively determine whether a tracer’s theoretical magnetic performance is realized experimentally [13,52,73].

**Core size and the effective magnetic volume.** The magnetic moment of a nanoparticle does not depend on its physical diameter, but on its effective magnetic diameter (*D*_m_) [30]. Single-core particles invariably exhibit a thin peripheral shell of disordered spins, caused by spin canting and crystal lattice irregularities that arose during synthesis, forming a “magnetically dead layer”. Consequently, the effective *D*_m_ is systematically smaller than the physical diameter, and it determines the Langevin curve and the resulting PSF [71,84]. To reach the theoretical saturation magnetization (~446 kA/m for phase-pure magnetite Fe_3_O_4_), synthesis routes must maximize crystallinity and suppress surface defects.

**Saturation magnetization and material composition.** Among iron oxide phases, magnetite (Fe_3_O_4_) is the preferred material for the MPI tracer, with a magnetic susceptibility roughly 1.5 times higher than maghemite (γ-Fe_2_O_3_), translating directly to higher signal per unit mass of iron [85,86,87,88,89]. The search for higher *M*_s_ has motivated the investigation of doped ferrite compositions (MFe_2_O_4_, where M = Mn, Ni, Co, or Zn), which redistribute magnetic cation occupancy across tetrahedral and octahedral sublattice sites in the spinel structure [85,86,87,88,89]. Zinc-substituted iron oxide nanoparticles (Zn-IONPs), for instance, have been shown to enhance *M*_s_ by approximately 37% and MPI signal intensity by up to 4.7-fold over standard iron oxide. Manganese ferrite is a further candidate of interest: its magnetocrystalline anisotropy constant is significantly lower than that of magnetite (3 kJ/m^3^ vs. 11 kJ/m^3^) [90], which would potentially accelerate Néel relaxation and improve the particle response to high-frequency drive fields in MPI. However, recent experimental data have contradicted these expectations [91,92], without a clear mechanistic explanation on how manganese ferrite falls short, indicating that significant additional research is required before Mn-ferrite’s theoretical advantages can be realized in practice.

**Size dispersity.** Monodispersity is a fundamental physical requirement for efficient MPI signal generation [13]. Because the Langevin function is nonlinearly size-dependent, a polydisperse ensemble behaves like an uncoordinated orchestra: particles with different magnetic moments and relaxation times respond “out-of-phase” to the drive field, causing PSF broadening and reduction in peak signal amplitude [93,94]. A standard deviation greater than ~10% in core diameter is sufficient to introduce measurable PSF broadening and signal heterogeneity across an imaging FOV [95].

**Magnetic (***K***) and shape anisotropy.** The magnetocrystalline anisotropy energy density directly governs τ_N_, as expressed in Equation (5). High anisotropy increases τ_N_, leading to signal smearing and PSF asymmetry, while low anisotropy enables faster magnetization switching, closer to the ideal adiabatic Langevin model [96]. Beyond crystalline anisotropy, particle shape introduces another layer of complexity called shape anisotropy. It has been reported that nanocubes achieved a 4.15-fold sensitivity improvement over “normal” spherical particles (VivoTrax) [77]. Currently, there is no scientific consensus on which shape is “perfect”: it is a balance between raw signal power (cubes) and image clarity (spheres).

### 5.2. Single-Core and Multicore Architectures: Design Logic

The relaxation wall narrows down the viable design space for single-core tracers considerably, but it does not represent an absolute ceiling on MPI tracer performance. Multicore and clustered architectures approach the problem from a structurally different angle, exploiting collective magnetic phenomena unavailable to isolated monodomain particles [78,79]. Each architecture carries a distinct set of physical strengths and failure modes, and an appreciation of both is necessary to understand the materials choices underpinning current high-performance tracer development.

**Single-core particles.** A single-core SPION represents the simplest possible signal source for MPI: a single crystalline magnetic domain producing a single magnetic moment [49], whose response depends only on particle volume, saturation magnetization, and relaxation behaviour within the classical Langevin framework. At the clinically accessible end, Ferumoxytol (Feraheme) carries the lowest regulatory barrier of any MPI tracer as an Food and Drug Administration (FDA)-approved intravenous iron supplement [80,81], but its 3–7 nm core places it well below the optimal Langevin window, resulting in peak MPI amplitudes nearly ten-fold lower than purpose-designed agents, such as the multicore VivoTrax Plus, and necessitating substantially higher iron doses for detectable signal-to-noise ratios. Its small size does, however, confer merit as a cell-tracing agent. In the commercial research sector, PrecisionMRX^®^ (Biosystems) provides a monodisperse 24–25 nm single-core platform with a PSF FWHM of 12.4 mT, offering good image quality that has made it a key benchmark for human-scale MPI feasibility studies [82]. At the research-grade frontier, the LS series (LS-1 and LS-008) represents the most widely cited high-performance single-core benchmark [83,95]: these 25–26 nm particles achieve a spatial resolution of 1.6–1.7 mm and a 3- to 5-fold signal intensity advantage over the commercial standard Ferucarbtran (Resovist, VivoTrax). The shape-engineered cubic iron oxide nanoparticles (CIONs-22), whose 4.15-fold sensitivity advantage over Ferucarbotran was noted above, have further demonstrated that this performance translates to biologically meaningful gains, enabling in vivo detection of fewer than 2500 bone mesenchymal stem cells [77]. At the most experimental end, FeCo@C alloy nanoparticles, 10 nm iron–cobalt alloy cores encapsulated within graphitic carbon shells, exhibit the highest M_s_ reported for any MPI tracer candidate, yielding 6- to 15-fold signal increase over clinical standards as Ferumuxytol and Ferucarbotran, derived from the intrinsically higher M_s_ of the FeCo alloy phase relative to any iron oxide [97].

**Multicore particles (nanoflowers and nanoclusters)**. Multicore architectures consist of multiple small magnetic crystallites (typically 5–10 nm in diameter), co-organized into a larger magnetic cluster, and held together by a polymer matrix or formed during co-precipitation through controlled aggregation. In these systems, it is important to distinguish between crystallite size, multicore cluster diameter, and hydrodynamic particle size, since each can affect MPI performance differently. The defining technical advantage of this design is the inter-crystallite magnetic coupling. Because the crystallites are closely packed, dipole–dipole and exchange interactions modify the collective magnetic behaviour. This can increase the effective anisotropy (K_eff_) of the cluster and promote cooperative magnetization transitions, in which multiple crystallites flip their magnetic orientation in a highly coordinated fashion, amplifying both sensitivity and peak height in the PSF [61].

Ferucarbotran (Resovist/VivoTrax^®^) is an iron oxide nanoparticle formulation with a hydrodynamic diameter of approximately 62 nm, composed of magnetite cores coated by carboxydextran, and has served as the foundational MPI benchmark. Its signal generation reflects the inefficiency imposed by its polydisperse architecture. It exhibits a bimodal magnetic core size distribution, with ~30% of cores at 25–30 nm and ~70% at 5 nm, the latter not magnetizing sufficiently to contribute to signal generation [98]. A magnetically fractionated variant, VivoTrax ^Plus^, enriches the larger-core fraction and shows improved MPI performance: a 2.4-fold higher signal and a 1.3-fold improved resolution compared to its predecessor [99].

MCP3 (Multicore Particle 3) represents a first-generation engineering response to the inefficiencies of Ferucarbotran. Transmission Electron Microscopy (TEM) shows that it has a multicore magnetic cluster diameter of approximately 32 ± 8 nm, and its synthesis is designed to maximize the proportion of clusters near the optimal effective magnetic diameter. MCP3 achieves approximately 5-fold higher sensitivity than Ferucarbotran in Magnetic Particle Spectroscopy (MPS) measurements, and a spatial resolution of ~2 mm in Bruker preclinical scanners: a meaningful but not transformative improvement [100].

Synomag-D (micromod Partikeltechnologie GmbH, Rostock, Germany) features a nanoflower substructure of ~5.5 nm iron oxide crystallites, coated with dextran to produce particles with a hydrodynamic diameter of 50 or 70 nm, and represents the current state of the art among commercially available multicore formulations [101,102]. It achieves an MPS FWHM of ~6 mT, and an MPI resolution of 1.0–1.6 mm, a 3.5-fold higher mass-normalized sensitivity than Ferucarbotran [103]. Its superior performance is generally attributed to its engineered internal architecture [104], although active debate persists over whether exchange coupling or disordered internal spin structures dominate the signal mechanism [105]. Despite its relative robustness, cellular internalization can still alter relaxation behaviour and introduce quantification errors in cell-tracking settings.

Perimag^®^, a larger dextran-stabilized multicore formulation built from ~5.5 nm crystallites with a hydrodynamic diameter of ~130 nm, also substantially outperforms Ferucarbotran, with a specific sensitivity of 29.49 mV/mgFe and an FWHM of 7.3 mT [23,106]. The combination of high sensitivity and fast dynamics makes Perimag particularly well-suited to high-iron-load cell labelling contexts, though its large hydrodynamic diameter restricts in vivo use, which will be evaluated further in the review [107].

**Architectural trade-offs and design selection principles.** Single-core tracers offer the closest correspondence to the idealized Langevin model [17], the narrowest achievable PSFs, and the most predictable size–performance relationships, all advantages that make them the preferred architecture when spatial resolution and quantitative fidelity are primary requirements [52,63]. Multicore architectures trade some of this theoretical elegance for practical considerations, such as ease of industrial production and robustness, achieving high sensitivity at effective diameters that would be inaccessible to single-cores [98,104]. The appropriate choice is, therefore, application-driven: single-core tracers for resolution-critical or quantitatively demanding tasks, and multicore nanoflowers for sensitivity-critical or environmentally robust requirements [35].

Future tracer development will likely move toward hybrid or application-specific designs rather than converging on a single universal formulation [47]. A compelling example is Superferromagnetic iron oxide nanoparticle chains (SFMIOs), assembled from individual SPIONs that self-organize into linear chain structures under the influence of an external magnetic field during synthesis [79]. When the applied field exceeds a dynamic coercivity threshold, magnetic reversal propagates cooperatively through the chain in a cascade-like switching event, generating an extremely steep *dM/dH* response and a correspondingly sharp and tall PSF, with experimental results reporting a 40-fold improvement in mass sensitivity and a 10-fold enhancement in spatial resolution compared to commercial benchmarks under equivalent scanner conditions [79]. Significant translational challenges remain, however: initial demonstrations were conducted in organic solvents, and developing aqueous, biocompatible formulations that preserve chain architecture under physiological ionic strength, pH, and protein adsorption conditions present a substantial materials engineering challenge.

### 5.3. Beyond the Core: How Coating and Biological Context Modulate MPI Signal

The surface coating contributes directly to the MPI signal by influencing the hydrodynamic volume. An increase in shell thickness, or the formation of coating-stabilized secondary aggregates, lengthens τ_B_ proportionally. For tracers where Brownian relaxation delays the SPIONs’ response, coating optimization is not only a biological question but also a magnetic one: a coating that is too thick can shift the dominant relaxation mechanism, widen the PSF, and reduce peak signal intensity. Conversely, a coating that is too thin leads to colloidal instability, aggregation, and the formation of large clusters whose Brownian dynamics are irrelevant to signal but whose size triggers immune clearance and vascular occlusion.

Targeting moieties grafted to the coating surface can further increase the hydrodynamic diameter, alter surface charge, and modify protein corona formation, all of which have downstream effects on circulation time, cellular uptake kinetics, and in situ MPI signal [108,109]. These interactions underscore that tracer design is a system-level engineering problem, and that optimizing the magnetic core in isolation is insufficient for the development of a high-performance, biologically validated MPI tracer. The subsequent sections address each of these components: magnetic core synthesis (Section 6), surface coatings (Section 7), and biomedical applications (Section 8), within this integrated design framework.

## 6. Nanoparticle Core Synthesis: Methods, Trade-Offs, and MPI Relevance

**Figure 4:**
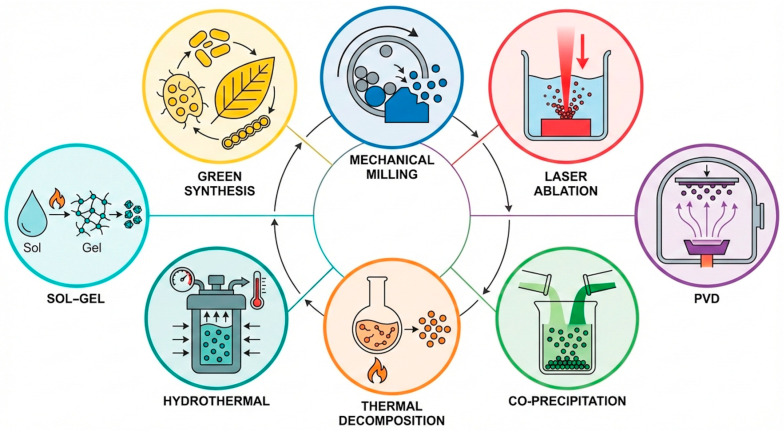
Synthesis pathways for magnetic nanoparticles. The choice of synthesis route—ranging from physical milling to precisely controlled chemical methods, like thermal decomposition—dictates the core size, monodispersity, and crystalline quality critical for MPI performance.

As established in Section 5, the magnetic core properties governing MPI performance, size, crystallinity, phase purity, and composition are not intrinsic to the material but are products of the synthesis route. Currently, the vast majority of MPI tracers utilize iron oxide-based nanoparticles, i.e., magnetite (Fe_3_O_4_) and maghemite (γ-Fe_2_O_3_), thanks to their high saturation magnetization and established biocompatibility [110,111]. Nanoparticle synthesis can be classified into “top-down” approaches, which physically or chemically break bulk materials down to the nanoscale, and “bottom-up” techniques, in which nanoparticles nucleate and assemble from molecular precursors [112]. A third emerging category, green synthesis, leverages biological processes to produce particles with inherently favourable biocompatibility profiles (Figure 4). The section below describes the most widely used methods within each category, discussing their key controllable parameters and their practical relevance to MPI tracer development; a comparative overview of working principles, advantages and limitations across all methods is provided in Table 1.

### 6.1. Mechanical Milling

Mechanical milling reduces bulk materials to the nanoscale through repeated high-energy impacts in milling equipment [113]. In ball milling, sealed vessels partly filled with grinding media are set in motion, causing the media to collide with the source material and generate impact and shear forces that progressively fracture it into nanoparticles [114]. Key parameters, such as mill type, rotation/vibration intensity, and duration, among others, govern the resulting size distribution, crystallite size, defect density, and, in some cases, phase constitution [115]. This method is well-suited for scale-up, employs low operational cost, simple infrastructures, and accommodates a broad range of metal nanomaterials. However, mechanical milling carries several notable drawbacks that limit its suitability for producing MPI tracers. Critically, the morphological control is inherently poor: the stochastic nature of the impacts makes it difficult to achieve defined particle shapes or narrow size distributions, and the resulting nanoparticles typically exhibit high polydispersity compared to those produced by chemical synthesis routes. This broad size distribution is particularly problematic for MPI applications, where tracer performance is highly sensitive to particle size uniformity. Beyond size control, the process frequently introduces structural imperfections, including rough surface morphologies, lattice defects, and contaminants originating from wear of the milling equipment, that can further degrade magnetic properties and biocompatibility. The high mechanical loads accelerate equipment wear, and the significant heat generation can trigger unwanted phase changes unless parameters are highly controlled [116].

### 6.2. Laser Ablation

In laser ablation, often referred to as Laser Ablation Synthesis in Solution (LASiS), a solid metal or oxide target submerged in a liquid is irradiated by a high-intensity laser [117]. The ablated material is directly converted into colloidal nanoparticle dispersion, typically without the need for chemical reduction agents or surfactants. By adjusting laser settings (power, wavelength, duration, …), it is possible to produce particles with tunable sizes, morphologies, and high chemical purity, compatible with both aqueous and organic liquid environments [118]. This method generates minimal chemical waste and provides fine control over particle size distribution. However, despite this tunability, laser ablation typically produces unwanted broader size distributions than bottom-up chemical synthesis methods due to the transient and non-equilibrium nature of NPs formation and nucleation in the liquid phase. This residual polydispersity, often manifesting as bimodal distributions in nanosecond ablation, can limit its suitability for MPI, where narrow size distributions are required for optimal magnetic response. Its primary limitations are lower productivity compared to other approaches, high energy input, and dependence on a sophisticated and expensive laser system, all of which can hinder industrial scalability.

### 6.3. Physical Vapour Deposition (PVD)

Physical Vapour Deposition (PVD) converts a solid source into a vapour phase and allows it to condense on a substrate, typically in a controlled, low-pressure inert atmosphere that limits contamination and provides precise control over growth conditions [119]. The source material is converted to the gas phase by an external energy input: in sputtering, plasma ions strike the target and eject atoms or small clusters; in evaporation, resistive or electron–beam heating generates atomic vapours; and in pulsed laser deposition, short laser pulses ablate the target. The resulting species travel through the low-pressure chambers and condense on the substrate, where nucleation and growth produce the NPs [120]. PVD yields high-purity products with uniform size distribution and allows for precise tuning of particle parameters, such as size, shape, and crystallinity. However, it requires specialized vacuum hardware and high-energy sources, increasing capital and operational costs. Nevertheless, for nanoparticle production, PVD methods can still exhibit variability in particle size due to nucleation and growth kinetics on the substrate, particularly when particles are harvested rather than grown in situ. This can introduce moderate polydispersity compared to solution–phase bottom-up methods, which may impact MPI performance if not carefully controlled.

### 6.4. Co-Precipitation

Co-precipitation is the most established and commercially implemented method to produce Fe_3_O_4_ nanoparticles [15]. The method typically involves alkaline precipitation of Fe^2+^ and Fe^3+^ salts, often in a 1:2 molar ratio, at temperatures between 70–90 °C under strong basic conditions (pH 9–14). It is attractive for manufacturing due to inexpensive and water-compatible reagents and straightforward scalability, and it naturally yields multicore aggregates [121,122,123,124]. However, reaction products are highly sensitive to synthesis parameters, such as the nature of the iron salts, Fe^3+^/Fe^2+^ stoichiometry, ionic strength, temperature, and pH, which together govern nucleation kinetics, growth rate, and aggregation behaviour, and, therefore, the final particle size distribution, morphology, and surface charge. Rapid kinetics in aqueous media often produce broad size distributions and relatively low crystallinity, uncontrolled oxidation or partial reduction during synthesis that can destabilize the Fe_3_O_4_ phase, resulting in particles that are highly reactive and prone to aggregation, requiring immediate surface stabilization. Through careful parameter optimization, however, co-precipitation can generate purpose-engineered tracers, such as MCP-3, that substantially outperform legacy materials [100,125].

### 6.5. Thermal Decomposition

Thermal decomposition is widely regarded as the benchmark route to produce highly monodisperse, single-core nanoparticles [71,126]. The method relies on the high-temperature decomposition of organometallic precursors in high-boiling organic solvents in the presence of coordinating surfactants. By decoupling the nucleation from the growth phase, and fine-tuning reaction parameters such as temperature, time, precursor concentration, types of surfactants, and solvent, it enables tight control over the size distribution, size, shape, crystallinity, and magnetic properties [122,127], including production with specific geometries, such as cubic Fe_3_O_4_ [128].

Post-synthetic treatments, for example, controlled oxidation, are frequently employed to convert non- or weakly magnetic secondary phases (e.g., wüstite, FeO) and to reduce the magnetic dead layer described in Section 5.1. Such processing increases the effective magnetic diameter and can substantially improve performance metrics.

Despite its unmatched reproducibility and property control, thermal decomposition carries significant practical and translational limitations. Elevated temperatures, high-boiling toxic organic solvents, and inert atmospheres make scale-up operationally and environmentally demanding. Furthermore, as-synthesized nanoparticles are typically hydrophobic due to their surfactant capping, necessitating additional ligand exchange or encapsulation steps to render them water-dispersible and biocompatible. These post-synthetic modifications enlarge the hydrodynamic size and, as discussed in Section 5.3, alter the relaxation dynamics that govern the MPI signal. Consequently, integrating thermal decomposition into clinically viable production requires careful consideration of scalability, reproducibility, and surface functionalization strategies [129].

### 6.6. Hydrothermal and Solvothermal

Hydrothermal and solvothermal routes occupy an intermediate position in the synthesis landscape, combining the aqueous compatibility and operational simplicity of co-precipitation with a level of structural control that approaches thermal decomposition. Reactions are performed in sealed autoclaves at elevated temperature and pressure over extended durations, favouring growth under near-thermodynamic control and reducing kinetic trapping [11,124]. The result is superior to standard co-precipitation with typically higher crystallinity, narrower size distributions, and saturation magnetization values closer to those achieved by thermal decomposition [105,122]. Particle properties can be further tuned by adjusting solvent composition, precursor concentration, and reaction time, enabling control over size and surface charge [130]. However, practical constraints remain: high-pressure equipment and long reaction times increase energy demand and complicate scale-up for industrial production [131]. Slower kinetics and sensitivity to autoclave-specific conditions can affect batch-to-batch reproducibility.

### 6.7. Sol–Gel

Sol–gel processing is based on the controlled conversion of molecular precursors (the sol) into a 3D gel network [132]. The process begins with the dissolution and activation of precursors in solvents (commonly alcohol–water mixtures), followed by hydrolysis and condensation (or polycondensation) that progressively build the oxide network. The gel is then dried to remove residual solvent, and subsequent heat treatment removes organic residues, promotes crystallization and stabilizes the oxide phase. The temperature profile (ramp rate, time, and atmosphere) governs the crystal growth, phase purity and defect chemistry, and, therefore, the final nanoparticle properties. This method offers strong compositional flexibility and straightforward control over surface chemistry. Its limitations include the tendency to form extended three-dimensional oxide networks that reduce the efficiency of dispersed nanoparticle production, potential contamination from byproduct reactions requiring additional purification, prolonged reaction times, and the use of toxic organic solvents [111,133].

### 6.8. Green Synthesis

Green synthesis, also frequently referred to as biological synthesis, represents an emerging paradigm that prioritizes environmental safety and inherent biocompatibility by exploiting natural biological processes [134,135]. It typically involves microorganisms or plant extracts to facilitate the reduction in iron precursors and the stabilization of the resulting magnetic nanoparticles (MNPs). Certain bacterial strains can produce highly stable magnetic structures: for instance, the *Bacillus cereus* strain HMH1 has been reported to produce iron oxide NPs, with an average size of 29.3 nm [136]. Fungi, such as *Aspergillus niger*, have been used to produce magnetic nanoparticles with dimensions of 15–18 nm [137]. Plant extracts represent another route; for example, banana peel has been used to coat co-precipitated SPIONs with polyethylene glycol (PEG), yielding monodispersed nanoparticles.

Among the most promising biogenic systems are magnetosomes produced by genetically modified magnetotactic bacteria, which are chains of magnetite crystals that represent a naturally occurring variant in the multicore chain architecture discussed in Section 5.2. These magnetosomes have demonstrated superior magnetic performances compared to Ferucarbotran and are being investigated as a living multimodal contrast agent for combined MPI, MRI and bioluminescent imaging [138,139]. In contrast to many other green synthesis routes, magnetosomes can also offer greater structural uniformity and reproducibility, making them particularly attractive candidates for MPI tracer development.

The primary advantage of the green synthesis routes is their low environmental impact and the high inherent biocompatibility of the resulting particles, which often require fewer post-synthetic modifications for physiological use. However, for many non-magnetosome green synthesis approaches, main limitations remain, including high polydispersity and morphological variability (with the consequent MPI performance penalties established in Section 5.1) and poor batch-to-batch reproducibility, which necessitates further research into standardized size–isolation techniques before these approaches can be considered for systematic tracer development [69,137,138].

## 7. Engineering the Nanoparticle Shell: Coating Strategies for In Vivo MPI Performance

**Figure 5:**
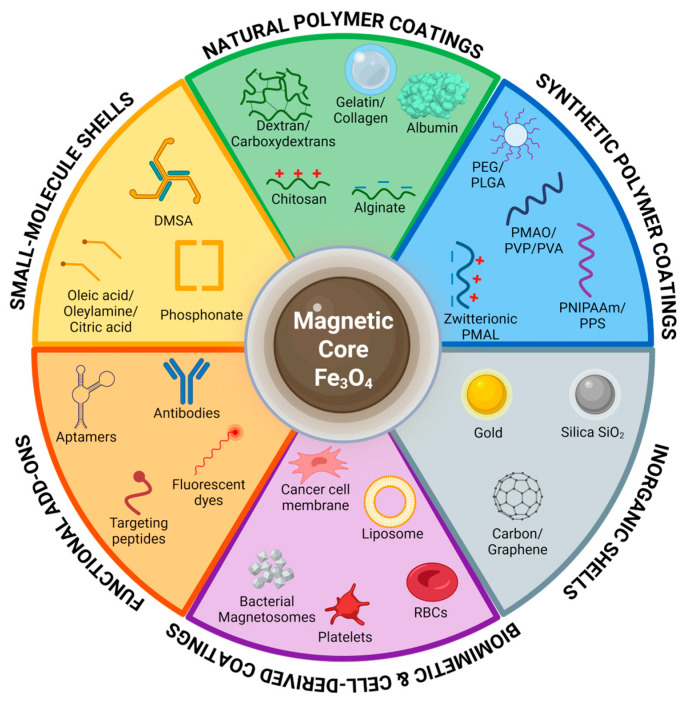
An overview of nanoparticle coating strategies for MPI applications. The wheel diagram summarizes the main classes of surface coating applied to Fe_3_O_4_ magnetic nanoparticle cores, organized into categories: small-molecule shells, natural polymer coatings, synthetic polymer coatings, inorganic shells, and biomimetic or cell-derived coatings. Functional add-ons, such as targeting peptides, antibodies, aptamers, and fluorescent dyes, are shown as a unique category, reflecting their use as modular modifications layered onto any primary coating. All categories are arranged at the same hierarchical level to represent the breadth of the available design options rather than an ordered coating sequence.

As introduced in Section 5.3, the surface coating is the primary determinant of colloidal stability in physiological media, biological fate, clearance kinetics, and magnetic relaxation behaviour [28,140], and coating selection is inseparable from the signal optimization strategy, since every decision at the coating level carries downstream consequences for MPI performance that cannot be corrected at the reconstruction stage [141]. Uncoated iron oxide cores are inherently unstable and tend to aggregate even under non-physiological conditions, a propensity that is further worsened in physiological media due to their high surface-to-volume ratio and strong magnetic dipole interactions. Surface coating directly addresses these colloidal and chemical limitations by providing steric or electrostatic repulsion, oxidation resistance, and a chemically addressable platform for targeting ligands or imaging reporters. However, coating does not resolve all failure modes; most notably, it does not prevent opsonization and subsequent reticuloendothelial system (RES) clearance upon systemic injection in vivo. These processes occur for coated and uncoated particles similarly; what the coating governs is not whether immune recognition takes place, but how rapidly and extensively it does, through its influence on coating chemistry, thickness, and surface charge. Coating design, therefore, determines not whether particles interact with the biological environment, but how, how quickly, and with direct consequences for circulation time, biodistribution, and, ultimately, MPI signal availability. This section explores the main coating classes in turn, focusing on their biological benefits and MPI-specific considerations. The main coating classes and representative examples discussed throughout this section are summarized in Figure 5.

### 7.1. Small-Molecule and Ligand Shells

Small-molecule and ligand shells represent the most basic layer of surface modification, consisting of discrete molecules that anchor to the iron oxide surface through defined coordination chemistries rather than forming a continuous encapsulating network. They are nearly universally present as the initial stabilizers on particles synthesized by thermal decomposition. Their primary limitation as a class is their thin protective layer, which leaves the core more vulnerable to long-term biodegradation in vivo compared to larger polymer architectures [142].

Oleic acid (OA) and oleylamine (OAm) bind to the iron oxide surface through carboxylate chelation and amine coordination, respectively, regulating reaction kinetics to confer precise control over core size, shape, and monodispersity during synthesis [143]. The resulting particles are exceptionally stable in non-polar organic solvents and maintain D_H_ very close to the crystallographic core size. From an MPI perspective, this near-core hydrodynamic diameter permits fast, nearly unconstrained Brownian rotation in organic phantom media and consequently near-ideal signal performance, establishing the baseline against which all subsequent Brownian relaxation time increases should be understood. This advantage is, however, strictly a laboratory artefact: the hydrophobic character of OA/OAm shells renders these particles incompatible with aqueous or biological environments, and direct in vivo injection would cause immediate aggregation and vascular clearance [144]. OA/OAm shells function exclusively as precursor coatings that must be displaced or encapsulated through phase-transfer protocols before any biological use.

Dimercaptosuccinic acid (DMSA) enables the most direct transition from hydrophobic precursor to stable aqueous suspension, displacing weaker surfactants through a ligand exchange and anchoring to the iron oxide surface via high-affinity oxygen-binding groups. The resulting coating provides colloidal stability in biological media for months, confers a strongly negative surface charge through terminal carboxyl groups, and demonstrates significant resistance to non-specific protein adsorption [145,146]. DMSA is non-toxic, and its residual carboxyl groups offer a versatile conjugation platform for dyes, drugs, and targeting ligands, and because it contributes a minimal increase in hydrodynamic diameter relative to the bare core, it imposes minimal Brownian relaxation penalties [147].

Citric acid (CA) and structurally related tricarboxylic acids bind to surface iron ions primarily through chelation, in which multiple carboxylate groups form coordinate bonds with surface iron ions in a stable ring-like configuration, with electrostatic interactions contributing secondarily through the influence of residual acidic groups on the particle surface charge, which enhances colloidal stability in suspension and electrostatic adhesion. CAs can be incorporated either through ligand exchange or directly during co-precipitation and hydrothermal synthesis [148]. CA-coated particles exhibit minimal hydrodynamic size increase and acquire a negative surface charge with negligible effect on the MPI signal, but in certain cell-labelling contexts, this charge has enhanced stem cell uptake [149,150].

Phosphonate ligands employ a phosphonic acid terminus to anchor to the nanoparticle surface with exceptional stability, providing a non-desorbing shell that resists removal under both aqueous and non-aqueous conditions [151]. These coatings act as a robust barrier against iron ion leakage, reducing the risk of oxidative stress in biological environments, and their surface enables flexible attachment of carboxyl, thiol, or amine functionalities [152].

### 7.2. Natural Polymer (Biopolymer) Coatings

Natural biopolymer coatings are derived from biological sources and valued for their inherent biocompatibility, biodegradability, and non-toxic profiles [153]. Their native functional groups (hydroxyl, amine, or carboxyl) allow direct conjugation of targeting proteins or drugs, without requiring intermediate linker chemistry.

Dextran is a hydrophilic polysaccharide of glucose monomers linked via α-1,6-glycosidic bonds that adsorbs onto nanoparticle surfaces under alkaline conditions through weak noncovalent interactions, including chelation and hydrogen bonding [154]. It provides high aqueous stability and prevents aggregation, although its noncovalent attachment is inherently weaker than covalent alternatives, and it does not confer full immune evasion when NPs are injected in vivo: rapid opsonization, and hepatic and splenic clearance through the RES remain characteristic features of dextran-coated particles. It is a semi-synthetic derivative of carboxymethyl dextran (CMD) that introduces carboxyl groups that strengthen surface binding and simplify chemical modification, and CMD is the coating underlying the most widely used clinically approved MPI-relevant formulations, including Resovist and Ferumoxytol [155]. The modest increase from a dextran shell means Brownian relaxation dynamics are not severely penalized, and the resulting negative surface charge can enhance cellular uptake in certain labelling contexts.

Chitosan is a cationic polysaccharide derived from chitin whose abundant primary amine and hydroxyl groups facilitate robust attachment to the iron oxide surface [156]. Although chitosan is protonated at the acidic pH required for its solubility (pH < 6), binding is not governed by simple electrostatic attraction; at this pH, the NPs’ surface is neutral or positively charged [157]. Instead, the primary stabilizing interactions are coordination/chelation of the amine and hydroxyl groups to surface iron atoms [158], and hydrogen bonding with surface-bound hydroxyls (Fe-OH) [159]. Electrostatic contributions are more often relevant during in situ co-precipitation (pH 9–14), where the strongly negative iron oxide surface (Fe-O^−^) may interact with any protonated polymer segments present [157]. Its defining biological characteristic is pH-dependent solubility: insoluble at neutral and basic pH, it becomes water-soluble under acidic conditions (pH < 6), potentially enabling triggered drug release in acidic tumour microenvironments [154]. It also possesses mucoadhesive and immunostimulatory properties that significantly influence biological interactions in vivo [160]. The strong cationic surface charge can increase non-specific cellular uptake, which is advantageous in cell-labelling protocols but may elevate cytotoxicity at higher concentrations.

Albumin coatings, typically based on bovine serum albumin (BSA) as an analogue to endogenous human serum albumin (HSA), are applied to form a uniform protein layer. This is achieved either through electrostatic adsorption onto charged cores or covalent conjugation, where amide bonds are formed between the protein’s primary amines and a pre-activated carboxylated nanoparticle surface [161]. This coating substantially minimizes immune recognition and opsonization, prolongs circulation time, and facilitates safer metabolic clearance compared to bare or dextran-coated cores [162]. The albumin layer neutralizes the surface charge, reducing non-specific binding to blood components, and provides a modifiable surface for drug loading or multimodal probe construction. The limitations include conjugation complexity and susceptibility to denaturation under high temperatures or extreme pH conditions that can be encountered within endosomal compartments.

Alginate, an electrolytic polysaccharide with carboxyl groups providing strong electrostatic adhesion to surface iron ions, confers a negative surface charge that prevents aggregation and is primarily applied in drug delivery contexts where biodegradability is a requirement [163]. Gelatin and collagen, both protein-based materials, are valued for their low immunogenicity and are frequently used in tissue engineering scaffolds and as tracer coatings in in vitro and ex vivo models, where their native cell-adhesion properties can be exploited [164,165].

### 7.3. Synthetic Polymer Coatings

Synthetic polymer coatings offer precisely controlled architectures and functional versatility that natural polymers cannot consistently replicate across batches. They are central to the pharmacokinetic engineering of MPI tracers intended for in vivo use because parameters, such as chain length (molecular weight) and grafting density, can be tuned to quantitatively modulate the hydrodynamic size and surface charge and, therefore, achieve specific blood half-lives.

Poly(maleic anhydride-alt-1-octadecene) (PMAO) is an amphiphilic polymer that wraps around hydrophobic core–ligand complexes via hydrophobic interactions with the pre-existing OA shell, providing an efficient phase-transfer route from organic to aqueous media without displacing the stabilizing inner ligand layer [85,166]. Its anhydride groups are frequently grafted with PEG to confer stealth properties, making PMAO-PEG encapsulation a two-layer strategy that preserves the monodispersity and magnetic properties achieved through thermal decomposition, while enabling aqueous biocompatibility.

PEG coating is the most widely applied strategy for extending nanoparticle circulation time [95,131,167,168]. It can be anchored via terminal functional groups (amines, thiols, or carboxyls) or through hydrophobic interactions when used in conjunction with an amphiphilic primary layer, such as PMAO [162,163]. By forming a dense hydration shell, PEG creates a steric “stealth” barrier that resists non-specific protein adsorption, substantially reducing recognition by the mononuclear phagocyte system and extending the blood half-life [169,170]. Hydrodynamic size increases with both the molecular weight (e.g., 5, 10, 20 kDa, or more) and the grafting density of the PEG chains, with a corresponding lengthening of the Brownian relaxation time and possible reduction of the MPI signal [83,171]. Though synthetic, it is a highly biocompatible polymer, and its easy renal clearance (molecular weights below ~30–40 kDa, with higher MW chains processed hepatically) minimizes the potential for accumulation and toxicity in the body, which is a crucial factor for the safety of any therapeutic agent. PEGylation shifts the surface charge toward neutrality, and terminal functional groups allow stable attachment of targeting antibodies, peptides, and fluorescent dyes. An important clinical caveat is the risk of immunogenicity upon repeated dosing: anti-PEG IgM antibodies can trigger the accelerated blood clearance (ABC) effect, reducing the efficacy of subsequent administrations [142].

Poly(lactic-co-glycolic acid) (PLGA) is an FDA-approved biodegradable polyester that typically encapsulates clusters of magnetic cores within a polymeric matrix or shell via double-emulsion or emulsion–evaporation methods [172]. PLGA constructs confer excellent drug-loading capacity for payloads, such as doxorubicin or siRNA [173], and at a mildly acidic pH (~6.5), characteristic of tumour microenvironments, the shell undergoes hydrolytic degradation and releases its payload [36]. The large hydrodynamic diameter of PLGA assemblies substantially lengthens the Brownian relaxation time and must be carefully matched in calibration phantoms when these platforms are used for MPI-guided drug delivery monitoring [36].

Polyvinylpyrrolidone (PVP) and polyvinyl alcohol (PVA) are hydrophilic synthetic stabilizers applied during alkaline co-precipitation or hydrothermal synthesis to provide aqueous stability and protection against over-oxidation, outperforming smaller surfactants, such as anionic sodium cholate, in this regard [114]. PVA is an uncharged, biocompatible, and biodegradable polymer that ensures uniform dispersion and reduces cytotoxic effects by shielding the iron core. PVP is an amphiphilic stabilizer that confers high colloidal stability in aqueous media and additionally improves nanoparticle crystallinity during co-precipitation by suppressing defect formation [109,174]. Their application is cost-effective and technically straightforward, making them common choices in large-scale synthesis workflows.

Among specialized synthetic architectures, zwitterionic polymers, such as 3-(dimethylamino) propylamine (PMAL), contain both positive and negative charged groups, providing high salt tolerance and stability across a broad pH range while minimizing non-specific binding, properties suited to high-selectivity targeted cell labelling [175]. Temperature-responsive polymers, such as poly(N-isopropylacrylamide) (PNIPAAm), can be engineered with a tunable lower critical solution temperature (LCST) to trigger drug release through collapse above threshold temperature in combination with magnetic hyperthermia [176]. Reactive Oxygen Species (ROS)-responsive polymers, such as polypropylene sulphide (PPS), enable drug release triggered by ROS in oxidative tumour microenvironments, allowing the theranostic tracer to respond to local disease state rather than an externally applied stimulus [177].

### 7.4. Inorganic Shell Coatings

Inorganic shells provide exceptional mechanical, chemical, and thermal stability that polymer coatings cannot match and are particularly well-suited to aggressive biological environments or applications requiring multimodal imaging integration [178].

Silane-based coatings are a surface functionalization strategy rather than a bulk encapsulation architecture. Organosilane coupling agents, bearing a surface-reactive trialkoxysilane terminus and a biologically functional end group, are grafted onto the nanoparticle surface, where hydrolysis and condensation reactions produce a thin covalently crosslinked siloxane layer. Unlike the thick continuous shells formed by Stöber silica deposition, this layer is nanometer-thin and does not substantially encapsulate the core; its purpose is to introduce stable, non-desorbing reactive handles, such as carboxyl, thiol, or amine groups, for the downstream conjugation of targeting ligands, dyes, or drugs [179].

Silica (SiO_2_) shells are most commonly applied through the Stöber process (in situ hydrolysis and condensation of tetraethyl orthosilicate, TEOS) or through microemulsion techniques that afford tighter size control [180,181]. The rigid silicate layer provides abundant surface silanol (Si-OH) groups, serving as highly stable covalent anchors for carboxyl, thiol, or amine functionalization, with minimal risk of ligand desorption, and the shell prevents iron ion leakage while maintaining excellent colloidal stability across a wide pH range, including acidic tumour microenvironment [109,182]. A key MPI consideration is that silica is diamagnetic: a thick shell reduces the mass-normalized saturation magnetization of the composite particle, an effect that must be factored into sensitivity calculations [181]. Silica also acts as a physical spacer preventing the iron oxide core from quenching optically active surface molecules, making it an important architecture for multimodal MPI/optical probe design [183].

Gold (Au) shells present a structurally distinct scenario due to the crystallographic lattice mismatch at the Fe_3_O_4_–Au interface, which sometimes necessitates an intermediate adhesion layer to ensure shell continuity [184]. Synthesized via seed-mediated growth or reduction in gold precursors onto the core surface, gold shells are highly biocompatible and non-immunogenic [185]. While the non-magnetic gold layer predictably reduces overall *M*s relative to bare cores, a noteworthy MPI-specific phenomenon has been reported: gold coatings can reduce magnetic anisotropy and increase differential susceptibility (*dM*/*dH*), which, counterintuitively, can strengthen the MPI signal compared to bare MNPs of equivalent iron mass [184]. This makes Au-coated particles a strategically relevant architecture for dual-mode MPI/CT [184,186].

Carbon-based coatings, including graphitic carbon shells, graphene layers, and carbon nanotubes, provide excellent chemical stability even under strongly oxidizing or acidic conditions, typically produced by chemical vapour deposition (CVD), pyrolysis of metal–organic framework precursors, or laser-induced photodecomposition [187]. When modified with secondary layers, such as polydopamine (PDA) or PEG, they resist immune detection and extend blood circulation [187]. Carbon encapsulation is the coating strategy of choice for FeCo alloy nanoparticles, as discussed in Section 5.2, whose reactive metallic surface would otherwise oxidize rapidly in biological media [188]. The practical limitations include high synthesis temperatures (typically above 800 °C), the need for density–gradient separation to manage broad size distributions, and the challenge of ensuring complete defect-free encapsulation at the nanoscale [188,189].

### 7.5. Biomimetic and Cell-Derived Coatings

Biomimetic coatings represent a conceptual departure from conventional surface chemistry: rather than engineering a synthetic material to approximate biological tolerance, these strategies borrow native biological structures directly to confer immune evasion and cell-specific targeting that purely synthetic coatings consistently fail to replicate with comparable fidelity [190].

Cell membrane coatings consist of nanoscale lipid bilayer shells derived from isolated fragments of red blood cells (RBCs), platelets, white blood cells (WBCs), cancer cells, or mesenchymal stem cells (MSCs), fused around the MNP core to produce a camouflaged core–shell structure [179]. Unlike whole-cell labelling, where particles are internalized into living cells, this process involves extracting the plasma membrane via hypotonic lysis and mechanical fusion of these isolated membranes onto the synthetic cores [191,192]. Membrane isolation is achieved through cell lysis and differential ultracentrifugation; fusion is achieved by mechanical extrusion, sonication, or electroporation of the isolated vesicles onto the synthesized cores [193]. Because of their biological origin, these particles present the full complement of membrane-associated proteins responsible for native cell identity, rendering them effectively invisible to immune recognition machinery [194]. Each membrane source confers a distinct biological targeting logic: RBC membranes exploit the CD47 and related self-marker proteins to extend blood circulation, making them highly relevant for perfusion imaging and angiography; platelet membranes confer affinity for collagen-exposed vascular lesion sites; cancer cell membranes exploit homotypic adhesion for tumour targeting; and hybrid membranes fusing components from multiple cell types can combine targeting and circulation properties in a single construct [195]. From an MPI standpoint, cell membrane coatings impose a relatively modest hydrodynamic diameter increase beyond the core, limiting the Brownian relaxation penalty in free suspension; upon cellular re-uptake, however, endosomal immobilization applies as with synthetic coatings [166].

Liposomes and lipid vesicles encapsulate MNP cores within a phospholipid bilayer aqueous vesicle, prepared by thin-film hydration or microfluidic techniques [196]. Liposomal encapsulation provides high stability in biological media and reasonable immune shielding, though less sophisticated than full cell membrane coatings [197]. The critical MPI constraint is that encapsulation typically results in large structures (50–500 nm), imposing significant Brownian relaxation penalties and substantially increasing the risk of rapid RES clearance due to size-dependent macrophage recognition. Interestingly, liposomes can be engineered with thermosensitive lipid compositions for triggered drug release or combined with plasmonic gold shells for trimodal MPI/MRI/CT imaging [196].

### 7.6. Functional Add-Ons: Targeting Moieties and Multimodal Reporters

Targeting peptides are typically attached by covalent bonding to terminal functional groups on the underlying polymer coating. An extensive combinatorial library exists, covering virtually any molecular target. Multiple peptides can be conjugated to a single particle for multivalent binding, and they add minimal mass and hydrodynamic volume, making them the least disruptive functional add-on from an MPI relaxation standpoint [198,199,200]. Their principal vulnerability is proteolytic degradation, which can strip targeting capability before the tracer reaches its intended site.

Antibodies and aptamers offer unmatched binding specificity and affinity for molecular targets but impose more significant physical consequences [201,202,203,204]. A single IgG antibody adds approximately 10–15 nm to the hydrodynamic diameter and considerable mass to the particle, both of which increase *τ*_B_ [205] and reduce the magnetic response of the tracer, a cost that must be weighed against the molecular specificity and multiplexing potential that antibodies confer [206]. Aptamers (nucleic acid sequences selected for high-affinity binding) represent a structurally more compact alternative that adds less hydrodynamic bulk than full IgG Ab [207]. Both conjugate classes are susceptible to degradation under the acidic conditions and enzymatic activity encountered within lysosomes and the broader endo–lysosomal pathway [166]. Multimodal imaging reporters, such as near-infrared organic fluorescent dyes (Cy7, Cy5.5, DiR), are covalently conjugated to the surface or physically loaded into the shell matrix [207,208,209]. Their role in an MPI context is not to enhance the MPI signal directly, but to provide complementary fluorescence imaging channels for cross-validation of tracer biodistribution [210]. Silica shell coatings are particularly well-suited to this integration because the diamagnetic matrix provides physical separation between the iron oxide core and organic fluorophores, preventing the fluorescence quenching that occurs when dyes are placed in close proximity to the metallic core [183].

### 7.7. Coating Selection as an Integrated Design Problem

The range of coating options highlights a core reality: there is no one-size-fits-all solution because coating design is inherently application dependent. The biological setting determines which relaxation processes dominate in vivo, which clearance routes prevail, and whether a given targeting approach is even relevant; the “best” coating can’t be chosen without first defining that context.

For example, a tracer intended for vascular perfusion imaging should emphasize stealth and long circulation while keeping hydrodynamic size increases to a minimum. By contrast, a tracer intended for cell labelling must be optimized to promote efficient endocytic uptake, where surface charge, hydrodynamic size, and the presence of targeting ligands are all relevant design parameters, so optimization shifts toward maximizing labelling yield, maintaining stability in endosomal conditions, and preserving cell health. A theranostic tracer that pairs MPI navigation with drug delivery or hyperthermia follows yet another design logic: here, the objective is often to engineer stimulus–responsive polymers that intentionally change hydrodynamic and magnetic behaviour in response to local pH, temperature, or ROS, turning environmental responsiveness into the goal rather than a drawback.

Across all these scenarios, the system matrix or calibration phantom used for image reconstruction should be aligned as closely as possible with the tracer’s true biological milieu. The next section considers each major application area in turn, showing how the coating and core design principles outlined above map onto application-specific tracer specifications, performance metrics, and translational limitations for vascular imaging, cell tracking, and nanoparticle-based theranostics.

## 8. Preclinical and Translational Applications

**Figure 6:**
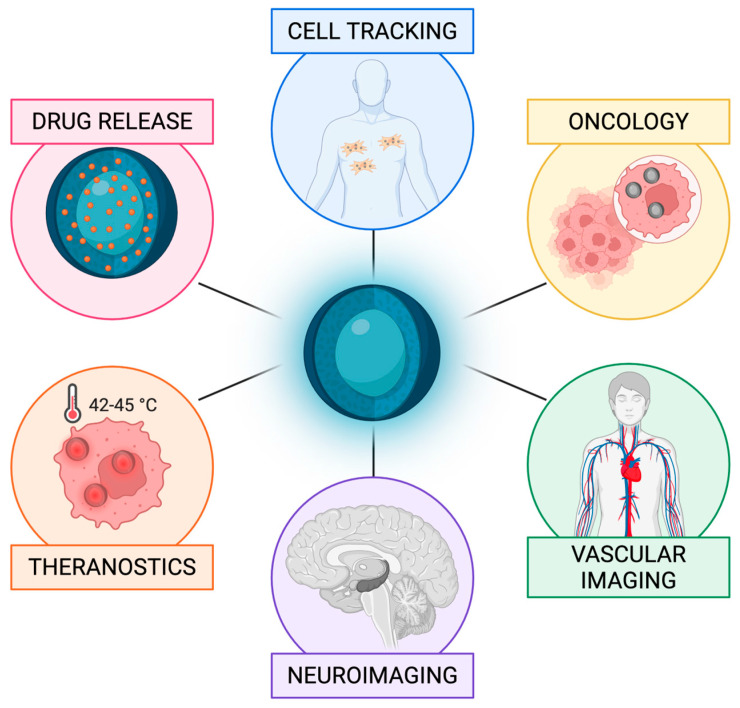
Preclinical and translational application areas of MPI. A pictorial illustration of the six application domains covered in this section: cell tracking, oncology, vascular imaging, neuroimaging, theranostics, and drug release monitoring. Each domain exploits a distinct aspect of MPI’s core capabilities: depth-independent quantification, real-time acquisition, and the shared physics of imaging and magnetic hyperthermia.

In this section, six application domains are discussed and illustrated schematically in Figure 6.

### 8.1. Cellular Imaging and Tracking

A major advantage of MPI for cellular imaging is that the signal scales linearly with the Fe content, independent of tissue depth or background. Preclinical studies this relationship holds with near-perfect fidelity (R^2^ ≥ 0.99) across concentration ranges, enabling the direct conversion of the signal intensity into the cell number, without correction factors or calibration curves that vary with anatomy [211,212]. This makes MPI very valuable for efficiently quantifying the cell numbers in various locations.

Although MPI is in principle highly linear and quantitative, the in vivo determination of cell numbers faces several biological limits. First, as labelled cells divide, the fixed initial iron load is partitioned equally among daughter cells [213]. The total iron mass within the region of interest may remain constant, but the signal per cell decreases with each division cycle, eventually falling below the voxel detection limit. This is particularly consequential for highly proliferative populations, such as Chimeric Antigen Receptor T (CAR-T) cells and cancer cell lines, where the signal underestimates true cell expansion. Second, MPI cannot distinguish between viable and apoptotic cells, and the signal persists until the iron is physically cleared by phagocytic cells [214]. A drop in signal may, therefore, reflect macrophage-mediated iron redistribution: for example, Kupffer cells in the liver or Tumour-Associated Macrophages (TAMs) sequester iron released from dying cells, generating hotspots that no longer correspond to the original therapeutic population [214]. Third, as established in Section 4.3, cellular internalization and confinement within endosomal vesicles restrict their Brownian relaxation [166]. Studies have documented a 20% decrease in magnetic performance upon endosomal encapsulation, and for Brownian-dominant tracers like Ferucarbotran, signal losses of up to 74% have been measured in high-viscosity environments [166]. Néel-dominant tracers, such as Synomag-D, whose signal arises from internal spin reorientation rather than physical rotation, are substantially more robust to this effect [17,101].

Against this background, the diversity of preclinical cellular tracking applications is significant. Optimized cubic iron oxide nanoparticles (CIONs-22) allow in vitro visualization down to approximately 30 cells/µL, with an in vivo detection threshold of around 2500 cells over at least seven days [77]. Mesenchymal stem cells labelled with Ferucarbotran or ferumoxytol have been tracked longitudinally following intravenous injection, revealing an initial entrapment in the pulmonary microvasculature before clearance and hepatic accumulation over twelve days [81,215]. Neural progenitor cells labelled with Resovist have been tracked for 87 days in the rat forebrain, with an in vivo detection threshold of 200 cells [215]. CLDN18.2-targeted CAR-T cells loaded via a protamine sulphate and heparin protocol (~3 pg Fe/cell) have been imaged migrating to gastric cancer xenografts over one week [216], while CD70-targeted CAR-T cells labelled with MegaPro, a formulation that has already completed Phase II clinical trials (NCT03407495), have provided the first quantitative spatial mapping of engineered T-cell distribution within glioblastoma mass versus the surrounding parenchyma [217]. In another study, dendritic cell migration has been detected to a limit of approximately 1000 cells, or 4.4 ng Fe—a detection capability that has pushed sensitivity to its current preclinical limit, and cannot be met by any other clinical imaging modality [218].

MPI also enables imaging of immune cells without direct labelling. A recent study showed that intravenously injected long-circulating Synomag-D–PEG tracers can be imaged after their phagocytosis by TAMs in situ, turning the inflammatory compartment of the tumour microenvironment (TME) into a signal source without any ex vivo manipulation [102]. The same principle extends to extracellular vesicles: coating the particles with glioma U-87 derived extracellular vesicles (EVs) facilitated the delivery of the nanoparticles into the tumour region within the brain, successfully crossing both the blood–brain barrier (BBB) and the blood–tumour barrier (BTB) to accumulate at metastatic sites in the brain, establishing MPI as one of the few modalities capable of quantifying barrier-crossing efficiency of biological carriers [219,220].

In summary, cell tracking with MPI has progressed to a point where it becomes a serious competitor to established modalities. The main remaining limitation is not sensitivity but interpretive validity: MPI cannot distinguish live from dead labelled cells, nor therapeutic cells from macrophages that have taken up their iron. For quantitative longitudinal studies where the signal is used to infer cell fate, complementary viability measures, such as bioluminescence imaging (BLI), remain essential [103].

### 8.2. Oncological Imaging and the Tumour Microenvironment

MPI has shown promise in the assessment of tumour microenvironments that goes well beyond simple tumour detection. The simplest exploited mechanism is the Enhanced Permeability and Retention (EPR) effect: because tumours develop leaky, poorly organized vasculature, long-circulating nanoparticles tend to accumulate specifically in the tumour environment over time, which can be quantified using MPI [61,221]. Using LS-008, a tracer with a ≈25 nm core diameter (≈26 nm by magnetic fitting) and a PMAO-PEG coating with a blood half-life of 105–108 min, researchers have mapped wash-in and retention dynamics in breast cancer xenografts (MDA-MB-231) over a 96 h window capturing, at peak accumulation six hours post-injection, the spatial heterogeneity of EPR-driven uptake that a single static scan would [22].

**Figure 7:**
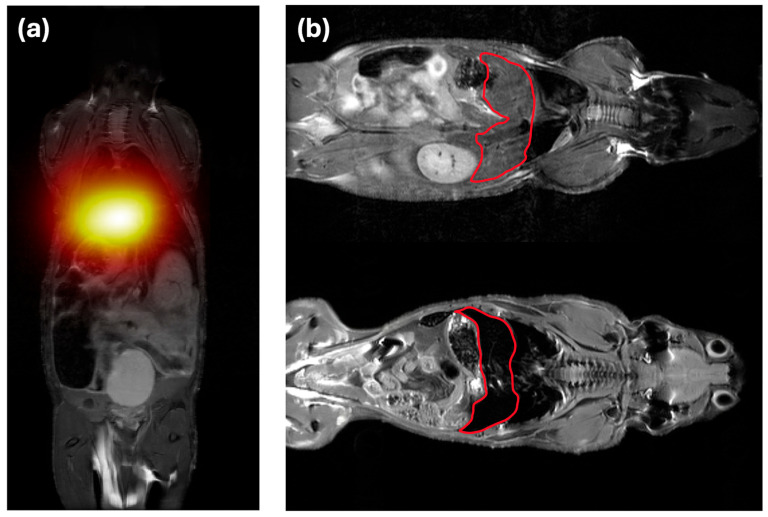
Complementary MPI and MRI of tracer biodistribution in a healthy mouse. Representative images acquired 30 min after intravenous injection of a VivoTrax (4 mg Fe/kg). (**a**) The MPI signal, overlaid on a structural post-injection MRI scan. The dominant signal is localized to the liver, consistent with the rapid hepatic uptake of dextran-coated particles by Kupffer cells via the reticuloendothelial system. (**b**) A T2-weighted MRI of the abdominal region before (**top**) and 30 min after (**bottom**) tracer injection. The liver (outlined in red) exhibits marked negative contrast enhancement post-injection, reflecting T2 shortening induced by nanoparticle accumulation. Together, the two modalities illustrate their complementary roles: MPI provides background-free, quantitatively absolute detection of tracer iron content but is limited in spatial resolution by PSF blurring. MRI delivers high-resolution anatomical detail but cannot directly quantify tracer concentration, as signal changes reflect proton relaxation alterations in the surrounding tissue rather than the iron mass per se. Adapted from [212].

However, purely passive accumulation is a limited strategy. Only 0.7–2% of administered nanoparticles are retained in the tumour via the EPR effect, while the vast majority is sequestered by the RES (predominantly liver and spleen) [222,223,224]. The resulting overwhelming liver signal often masks the weaker tumour signal [102,225]. This is illustrated in Figure 7, which shows representative MPI and MRI images of the abdominal region in a healthy mouse 30 min after intravenous injection of VivoTrax at a dose of 4 mgFe/kg: the dominant MPI signal localizes to the liver, reflecting rapid Kupffer cell uptake, while the corresponding MRI confirms marked negative contrast enhancement in the same region. The collocation underscores the background-free, quantitatively absolute iron detection of MPI, but the very sensitivity that makes it powerful also captures off-target RES accumulation with equal fidelity, obscuring weaker signals from intended targets. In clinical applications, the EPR effect is also substantially weaker and more variable in human tumours than in the murine xenograft models, a significant barrier to clinical translation of passive targeting strategies [206].

Active tumour targeting directly addresses specificity and penetration with quantitative benefits that are substantial and measurable [108,210,226]. RGD-conjugated (the most common peptide motif responsible for cell adhesion to the extracellular matrix) iron oxide nanochains targeting angiogenic vessels in 4T1 breast tumours achieved a 17.2% uptake of the injected dose per mg of tumour tissue versus 9.2% for non-conjugated controls: a 1.87-fold improvement attributed to receptor engagement [227]. More strikingly, CREKA-functionalized iron oxides targeting the fibrin–fibronectin complex of the tumour stroma yielded MPI signals 5.7-fold higher than the commercial standard VivoTrax and 1.5-fold higher than non-targeted iron oxides in the same breast cancer model [91]. A critical additional finding was that active tracers distributed uniformly throughout the tumour mass four hours post-injection, whereas passive EPR accumulation remained confined to the tumour rim, a localization that would be therapeutically insufficient for hyperthermia [91]. The spatial quality of accumulation, and not only its magnitude, emerges as a feature that passive targeting fails to provide. This principle is further amplified by magnetic guidance: combining lactoferrin receptor-targeted nanoparticles with external permanent magnet steering in brain glioma models produced tumour accumulation significantly higher than either targeting strategy alone, demonstrating that the physical and biological mechanisms are synergistic rather than redundant [228].

A further development in oncological MPI is the engineering of tracers whose signal itself encodes biological information about the microenvironment, transforming MPI from a spatial mapping tool into a molecular sensor [229]. The mechanistic foundation is the relaxation physics established in Section 4: if a tracer’s Brownian rotation is constrained by cluster assembly, its signal is suppressed, but if the cluster disassembles in response to a biological trigger, the signal recovers. A pH-responsive PdFe alloy tracer releases iron ions at the acidic threshold of the tumour microenvironment (pH 6.4), generating a signal change whose correlation with intratumoural pH reached R^2^ = 0.90 at 60 min post-injection [229]. A ROS-responsive tracer disassembles in the presence of oxidative stress at concentrations as low as 0.05 mg/kg Fe, yielding a 27-fold signal increase in injured versus healthy tissue [229]. ATP-responsive probes targeting the metabolic signature of the TME achieve signal-to-background ratios 14.3-fold higher than non-responsive controls in metastatic lymph nodes [229].

Subtraction-enhanced MPI (SE-MPI) exploits the different temporal kinetics of tumour-responsive versus liver-accumulated tracers: because the liver signal is relatively stable over time, while the tumour signal evolves dynamically in response to the local microenvironment, subtracting late-phase from early-phase images mathematically isolates the tumour-specific component [225]. This approach improved tumour-to-liver ratios from 0.42 before subtraction to 5.66 after 120 min: a 13.5-fold enhancement in diagnostic specificity without modifying the tracer itself [225]. Alternatively, focused FOV acquisition restricts the imaging volume to the tumour region alone, effectively excluding the liver from the measurement window [102].

Sentinel lymph node biopsy represents the closest application to near-term clinical implementation [230]. The tracer design requirement for lymphatic applications is distinct from intravascular imaging: hydrodynamic diameters between 50 and 150 nm are needed to ensure interstitial drainage into lymphatic channels without either entering capillaries (below 50 nm) or remaining immobilized at the injection site (above 150 nm), with carboxydextran coatings, such as those used with Magtrace [231].

Moving these preclinical results toward clinical protocols will require both human-scale scanner development and a deliberate re-evaluation of targeting strategies in more physiologically representative tumour models.

### 8.3. Vascular and Perfusion Imaging

While MRI’s strength in vascular imaging lies in its high spatial resolution and superior soft-tissue contrast, these properties become limiting when the clinical question shifts from anatomy to dynamics [22,29,81]. Blood flow is rapid, and the haemodynamic parameters, such as flow velocity, transit time, and perfusion volume, are encoded in tracer concentration curves that evolve within seconds or less. MPI’s capacity for real-time imaging is particularly valuable here: by continuously sweeping the FFP along a pre-designed trajectory, MPI can capture the fast temporal dynamics [195,232,233]. The haemodynamic parameters that MPI can extract are substantive. From concentration–time curves generated by 3D acquisition, researchers have derived relative cerebral blood volume (rCBV), relative cerebral blood flow (rCBF), relative mean transit time (rMTT), and relative time-to-peak (rTTP), with values reported as equivalent to those obtained by MRI but in real time [195]. Blood flow velocities in the inferior vena cava have been measured at 4.8 ± 1.1 cm/s, in close agreement with MRI values of 4.0 ± 1.5 cm/s [234], while phantom systems have validated velocity measurements up to 64 cm/s [235]. MPI has resolved vascular stenoses as small as 2 mm [24], and the most sensitive human-scale brain imager prototypes have demonstrated a detection limit of 263 pmol Fe/mL, sufficient to detect perfusion deficits [236].

The pharmacokinetic requirements for vascular imaging are specific and non-trivial. Standard clinical iron oxide formulations, such as Resovist, with a blood half-life of approximately 30 min in mice, are inadequate for anything beyond first-pass imaging [27]. Dynamic angiography requires tracers that remain in the bloodstream long enough for multiple measurements, which demands PEGylation strategies that physically shield the nanoparticle surface from opsonization. The optimized tracer RL-1, a 22 nm single-core particle with PEG–silane coating, achieves a half-life of 6.99 h, nearly 12-fold that of Ferucarbotran, by maintaining a PEG molecular weight of 20 kDa at 18.8% surface loading density [83]. The difference between a tracer that disappears in 35 min and one that circulates for seven hours is crucial in determining whether serial vessel lumen measurements, longitudinal perfusion monitoring, or slow-acquisition high-resolution angiography are feasible at all. Tracer selection determines the outcome of the experiment: optimized multicore particles, such as MCP-3, with narrow PSF and signal intensities 4 to 5 times higher than Resovist, have enabled measurements of pulsatile vessel lumen diameter changes in the inferior vena cava and abdominal aorta that were unreachable with standard MPI–Resovist protocols [237,238].

**Figure 8:**
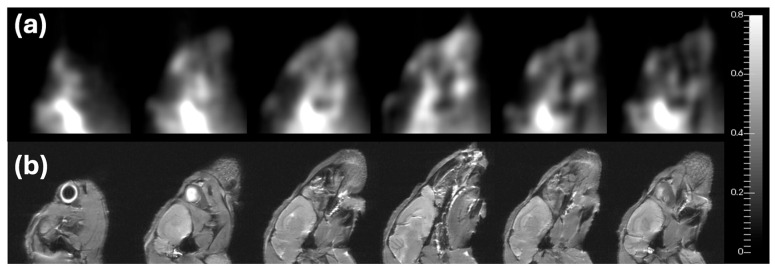
A tomographic MPI reconstruction of the mouse head vascular system. Sagittal slices acquired 30 min after intravenous injection of a PEGylated Synomag-D SPION tracer (core diameter: 50 nm; dose: 2.5 mgFe/kg), using x-space reconstruction. (**a**) MPI signal maps, showing tracer distribution within the major cerebral and cervical vasculature. The visible blurring reflects the convolution of the true tracer distribution with the finite point spread function (PSF) inherent to x-space reconstruction, which limits the resolution of fine anatomical structures. The signal intensity is displayed in arbitrary units (a.u.). (**b**) Corresponding T2-weighted MRI reference images acquired without contrast agent, providing a soft-tissue anatomical context. Images adapted from a presentation by Dr. Andre Bongers at the Australian National Imaging Facility (NIF) Symposium 2022, Adelaide, AU.

Multi-colour MPI represents the current technical frontier in vascular imaging [239]. Different nanoparticle formulations, or the same formulation in different physical environments, generate distinct harmonic spectra or relaxation time constants that can be deconvolved into separate image channels [240]. Perimag, Nanomag-MIP, and VivoTrax are discriminable by their τ values of 3.02, 2.67, and 4.15 ms, respectively [241,242], allowing them to be assigned distinct “colours” in the same imaging volume. Because Brownian relaxation is sensitive to the viscosity of the surrounding medium, as established in Section 4, a single tracer generates distinct signals, depending on whether it is suspended in flowing blood or immobilized in a coagulated clot [241]. This has been used to simultaneously visualize active haemorrhage and static haematoma in intracranial bleeding models, distinguishing haemorrhagic from ischaemic events in real time. This is usually a diagnosis that requires separate imaging with different modalities [243]. The ratio of the fifth to third harmonics (A_5_/A_3_) serves as a quantitative environmental sensor: for example, for liquid Synomag–COOH, this ratio is 12.3%, rising to 25% for cell-internalized particles [244]. The complementary relationship between MPI and MRI in vascular imaging is illustrated in Figure 8, which shows a tomographic x-space reconstruction of the mouse head vascular system acquired 30 min after intravenous injection of PEGylated Synomag-D tracer. The MPI signal maps the distribution of tracer within the major cerebral and cervical vasculature, while the co-registered T2-weighted MRI provides soft-tissue anatomical context without contrast agent. The spatial blurring visible in the MPI images is a direct consequence of convolution with the finite tracer PSF.

Multi-colour channel leakage, the bleeding of one tracer’s signal into another’s, limits the spectral resolution of multi-colour systems and requires extensive pre-characterization of each tracer in the specific temperature, viscosity, and binding environments it will encounter in vivo [240,245]. These are not reasons to discount the approach but specifications for the work that remains to be done.

### 8.4. Neuroimaging

The brain presents MPI with its most demanding target and its most instructive test case. Two protective barriers define the challenge: the BBB restricts which tracers can reach cerebral tissue at all, demanding either active transport mechanisms, vesicle-mediated delivery, or pharmacological opening before imaging can begin. The skull attenuates optical signals entirely and introduces susceptibility artefacts that degrade MRI in bone-adjacent structures, but is entirely transparent to the oscillating magnetic fields that drive MPI [219].

This advantage is clearest in acute stroke, where the speed of diagnosis directly determines tissue salvage. MPI detects ischaemia not by waiting for the secondary biological consequences (cytotoxic oedema, detectable by diffusion-weighted MRI or T2-visible lesion formation) but by directly measuring the absence of tracer arrival in the ischaemic territory. When a vessel occludes, the blood-borne tracer simply does not appear [246]. This produces a quantifiable perfusion deficit within seconds of tracer injection, at a temporal resolution of 21.5 ms per frame compared to 177 ms per frame for 2D DSC-MRI, an 8-fold improvement [195].

Intracranial haemorrhage presents a different diagnostic problem: not the absence of flow but its pathological persistence and the coexistence of active bleeding with static clotting. Multi-colour MPI, whose mechanism was established in Section 8.3, addresses this directly. In traumatic brain injury models, LS-13 tracers with blood half-lives of four to six hours allowed detection of haematoma immediately post-injury and at day three [31].

The BBB imposes a categorical constraint on neurological disease imaging beyond acute vascular events. Standard SPIONs injected systemically cannot cross an intact BBB, which means any MPI application targeting brain parenchyma, neurodegeneration, neuroinflammation, or tumour infiltration requires a delivery strategy that passes the barrier or a pathological condition in which it is already compromised. Three approaches have been validated preclinically. Receptor-mediated transcytosis using lactoferrin-conjugated nanoparticles, introduced in Section 8.2 for brain tumour targeting, exploits the brain’s endogenous iron transport machinery to achieve intracranial accumulation, with a detection limit of 1.1 ng Fe at SNR 3.9 in glioma models, a sensitivity that MRI T2* imaging could not match due to susceptibility signal voids [219]. As described in Section 8.1, iron-loaded EVs harvested from tumour cells successfully localized to the brain metastases, while bare SPIONs showed no intracranial signal, demonstrating both EV-dependence and lesion-specificity of barrier crossing. Focused hyperthermia-mediated barrier opening represents a third strategy: MPI detected nanoparticle retention in the targeted brain region for over 72 h post-opening [247].

Neurodegenerative disease represents the early frontier of MPI neuroimaging. Human neural progenitor cells have been tracked in the rat forebrain for 87 days at a detection threshold of 200 cells with signal linearity maintained at R^2^ = 0.99, establishing the quantitative framework for monitoring cell-based therapies in Parkinson’s and Alzheimer’s disease [215]. Antibody-functionalized tracers targeting amyloid–beta aggregates have been proposed for Alzheimer’s imaging, exploiting the high binding affinity of anti-Aβ antibodies to generate a localized signal in plaque-dense regions, but for now, this application remains at the tracer development stage [142,248].

Functional MPI uses changes in cerebral blood volume as an indirect readout of neural activity [249]. In hypercapnia-stimulated rat models, fMPI detected a 10% signal change with a Contrast-to-Noise Ratio (CNR) of 50 at a clinical iron dose of 7 mg Fe/kg [233].

For translation to humans, the main bottleneck is spatial resolution rather than sensitivity. A human head MPI scanner (25 cm bore) has been reported to achieve ~5–6 mm resolution in-plane and ~26 mm axially [236]. That is enough to distinguish large brain regions, but not fine structures, like cortical layers. Improving resolution likely requires stronger gradient hardware [66], tracers engineered to sharpen the effective PSF, and improved image reconstruction [79]. Deep-learning-based reconstruction has already reduced full-width-at-half-maximum by 43.6% in phantom studies [250].

In neuroinflammation and immune cell tracking, MPI produces hotspot signals, avoids bone-related artefacts, and remains linear. For example, anti-Ly6G-conjugated SPIONs designed to bind neutrophils can produce CNR values of ~8–12 between inflamed and healthy tissue [251], without the susceptibility-related signal voids that can be difficult to distinguish from haemorrhage or bone in MRI, and without the radionuclide half-life and cumulative radiation constraints that limit longitudinal PET tracking to hours or days. MPI tracer can remain stable for weeks to months, enabling long-term monitoring of processes such as post-stroke inflammation. While this apparent persistence may suggest long-term stability in vivo, it reflects compartment-dependent pharmacokinetics rather than resistance to biodegradation. SPION tracers are fundamentally biodegradable and, following cellular internalization, are typically sequestered in endolysosomal compartments where they undergo gradual metabolic degradation. The released iron is subsequently incorporated into the body’s endogenous iron pool and reused in physiological processes such as haemoglobin synthesis [46].

A key distinction, therefore, arises between systemic pharmacokinetics and local signal persistence. In the circulation, most SPION tracers exhibit relatively short blood half-lives in the order of 1–7 h before rapid sequestration by the reticuloendothelial system (RES), primarily in the liver and spleen. In contrast, once localized within brain tissue, either through barrier crossing or direct implantation, clearance is significantly slower due to the limited phagocytic and metabolic turnover capacity of the CNS parenchyma. This enables MPI to track labelled cells or tracer deposits over timescales of weeks to months [215].

However, this prolonged signal persistence introduces important interpretive considerations. In proliferative cell populations, the MPI signal per cell may decrease over time due to iron dilution during cell division [103]. In addition, iron released from dead or dying-labelled cells can be scavenged by resident macrophages or microglia, leading to so-called “bystander labelling.” This can result in a persistent MPI signal that no longer corresponds to the originally labelled therapeutic or target cell population [103]. Consequently, while MPI provides highly quantitative measurements of iron mass, interpretation in longitudinal neuroimaging studies must account for these pharmacokinetic and biological redistribution effects.

### 8.5. Theranostics: MPI-Guided Therapy

Theranostics, the integration of diagnosis and therapy within a single agent and workflow, has been a conceptual ambition of nanomedicine for two decades. The AMF used to produce the MPI signal is the same type of field used to heat magnetic nanoparticles in hyperthermia, differing primarily in frequency and amplitude [35,252]. The same tracer that images a tumour can be used for treatment, without changing agents or moving the patient to a different device. The key safety question is whether MPI can heat only the target while avoiding organs like the liver, where nanoparticles often accumulate [253].

Strong preclinical evidence suggests it can, when the selection gradient is used correctly [33]. With the gradient off, the drive field acts everywhere, so nanoparticles that have accumulated in the liver can heat, as well, causing severe off-target thermal injury. With the gradient on, particles outside the field-free region are magnetically saturated and effectively “switched off”, so they do not respond to the drive field. Therefore, heating is confined to the FFR. How tight the “heat spot” is depends mainly on gradient strength: a 2.3 T/m selection gradient produces a heating region of ~7 mm (FWHM), while 7 T/m narrows it to ~2.35 mm [66], enabling millimetre-scale control of thermal deposition at depth. Imaging and therapy are also operationally separated by using different field settings and improving safety: one study used ~20 kHz and 20 mT for imaging, and ~354 kHz and 13 mT for therapy [33]. This separation blocks unintended heating during imaging and minimizes the imaging signal during therapy.

At high performance, the limiting factor becomes the tracer design, not the scanner. Heating efficiency varies dramatically across formulations. For example, bimagnetic core–shell nanocubes (zinc-doped magnetite core with cobalt ferrite shell) have reported specific absorption rates around 10,600 W/g, compared with 4206 W/g for uncoated ~70 nm cubes, a 2.5-fold gain attributed to core–shell engineering that reduces surface-related magnetic losses [254]. Targeted systems (e.g., IO-CREKA) have been used to maintain tumour temperature in the 42–45 °C range typical of apoptosis-inducing hyperthermia, with the added benefit of uniform distribution as seen in Section 8.2: active targeting spreads the tracer more uniformly through the tumour rather than limiting it at the rim, which helps deliver heat to the interior [91,187]. MPI strengthens this approach because it can quantify not just whether the tracer is present, but where it is and if the concentration and distribution look sufficient before therapy begins.

Precision heating may also matter immunologically. In gastric cancer models, MPI-guided hyperthermia with CLDN18.2-targeted nanoparticles promoted immunogenic cell death and antigen release, shifting the tumour toward a more immune-active state [255]. When combined with anti-PD-1 therapy, tumour weight reductions were significantly greater than either treatment alone (*p* < 0.0001 vs. PBS control), depending on targeted, spatially controlled heating [255]. This positions MPI-guided hyperthermia as a potential adjuvant to immunotherapy rather than only a thermal ablation technique.

MPI also supports real-time drug release monitoring. As established in Section 8.2, stimuli–responsive nanocarriers suppress MPI signal through inter-particle interactions when clustered; cluster dispersal in the TME recovers particle mobility and restores signal proportionally. In one study, the MPI signal correlated with cumulative doxorubicin released at R^2^ = 0.991, meaning the image acts as a real-time quantitative readout of local drug dosage [36]. The gradient ON/OFF experiments captured the core point: the physics that creates the image can also enforce safety by confining therapy to the target. The remaining hurdles are mainly practical—scaling hardware, optimizing safe human field parameters, qualifying tracers, and proving benefit in clinical trials.

### 8.6. Practical and Safety Constraints in Preclinical and Clinical MPI

Translating MPI from preclinical proof-of-concept to clinical practice requires navigating a set of practical and biological safety constraints that operate at every level of the imaging chain, from tracer administration to scanner operation and that apply, to varying degrees, in both animal and human settings.

At the tracer level, the primary safety concern is iron dose. Purpose-engineered research tracers achieve detection at nanogram-to-microgram iron quantities, but clinically available formulations with suboptimal MPI performance, such as Ferumoxytol, require substantially higher doses to generate usable signal-to-noise ratios. While intravenous iron is generally well tolerated, high-dose administration carries risks of anaphylactoid reactions, oxidative stress from free iron release, and iron overload in subjects receiving repeated administrations. Regulatory qualifications of any new tracer must therefore address not only magnetic performance but acute and chronic toxicity, biodistribution, clearance kinetics, and metabolic fate of the iron load. Currently, no purpose-built MPI tracer has completed a full clinical regulatory qualification.

At the biological level, RES sequestration is the dominant practical limitation shared across all in vivo MPI applications. Regardless of coating strategy, a substantial fraction of any systemically injected nanoparticle dose is captured by Kupffer cells in the liver and macrophages in the spleen within minutes of the injection. In preclinical settings, this produces an overwhelming liver signal that frequently masks weaker signals from target tissues, a problem partially addressable through SE-MPI subtraction or focused FOV acquisition, as discussed in Section 8.2, but not eliminable through tracer engineering alone. In clinical settings, where EPR-driven tumour accumulation and barrier-crossing efficiencies are lower than in murine models, RES competition will be proportionally more severe. For cell tracking applications, macrophage uptake of iron released from dying labelled cells creates signal hotspots that no longer correspond to the therapeutic population of interest, imposing a fundamental interpretive limit on signal-to-cell-number inference that viability reporters must complement.

At the scanner level, human MPI operation is bound by two hard physiological safety limits. The first is peripheral nerve stimulation (PNS): the oscillating drive field induces electric currents in tissue, and above approximately 7 mT at 26 kHz, the stimulation of peripheral nerves becomes perceptible and potentially harmful [256]. This constrains drive-field amplitude in human systems and limits how strongly the tracer can be excited, directly reducing signal-to-noise ratio and image quality relative to preclinical scanners that operate without this restriction. Increasing excitation frequency while reducing amplitude is the proposed engineering workaround, but the optimal frequency–amplitude combination for human use remains an active area of investigation and will require regulatory validation. The second limit is the specific absorption rate (SAR): prolonged high-frequency AMF exposure deposits energy in tissue, and cumulative thermal effects must be managed within safe exposure guidelines, particularly for theranostic applications where sustained field application is intended to produce local heating. In this context, the ON/OFF gradient strategy described in Section 8.5 is not only a targeting mechanism but also a safety architecture: saturating off-target particles prevents unintended systemic heating and is likely to be a regulatory requirement for any clinical hyperthermia protocol.

In preclinical settings, the above constraints apply in attenuated form, PNS thresholds in small animals differ from those in humans, and iron doses that are safe in mice may not scale proportionally, but their practical consequences are real. Gradient strengths achievable in small-bore preclinical scanners cannot be replicated in human-scale systems, meaning that resolution and sensitivity benchmarks established in rodent models represent an optimistic ceiling rather than a clinical baseline. The field-free region geometry, coil design, and thermal management all change nonlinearly with bore size, and performance characterization in phantoms does not substitute for validation in living tissue across the full range of intended operating conditions.

Taken together, these constraints define a practical hierarchy for clinical MPI development: indications requiring lower iron doses, single administrations, and anatomically confined imaging volumes face the fewest cumulative barriers, while whole-body oncological theranostics, where high doses, repeated administrations, systemic distribution, and sustained field exposure converge, represent the most demanding regulatory and safety environment. Matching scanner design, tracer choice, and clinical indication from the outset, rather than optimizing each independently, is therefore not only a scientific recommendation but a translational necessity.

## 9. Conclusions and Future Perspectives

This review started with a simple observation: every clinical imaging modality makes a trade-off if you want deep-tissue imaging that is both radiation-free and truly quantitative. MPI was discussed as a focused solution to that specific gap, and not as a universal replacement for MRI, CT, or PET. MPI has matured from a physics demonstration into a modality with a defined clinical identity. Its signal originates exclusively from iron oxide tracers against essentially zero biological background, yielding signal-to-background ratios above 1000:1, and its linear relationship between iron mass and MPI signal (R^2^ ≥ 0.99) does not degrade with depth, enabling absolute quantification without the correction steps other modalities require. These advantages translate into concrete biological capabilities: detecting small cell populations at depth, identifying ischaemic injury earlier than diffusion-weighted MRI, measuring drug release quantitatively in real time, and delivering spatially confined hyperthermia that spares off-target organs. They are, however, unevenly matched to clinical needs: MPI is strongest where quantitative, real-time tracer detection matters more than anatomical detail, and it should not be positioned to compete with MRI or CT on structural grounds.

Three interdependent gaps define the remaining distance to routine clinical use. The tracer gap is the most immediately solvable: the physical design space is well characterized, but no formulation yet combines the magnetic performance of purpose-built research tracers with the scalability, pharmacokinetic profile, and regulatory qualification that clinical deployment requires. Ferumoxytol carries the lowest regulatory barrier but performs well below the optimal MPI window; MegaPro has advanced further clinically, but does not cover the full application range. Closing this gap demands indication-driven development—specifying the biological environment first and designing the tracer around it, rather than optimizing against phantom benchmarks. The scanner gap is an engineering problem with known solution pathways—higher-performance gradient arrays, advanced reconstruction including deep learning, and drive-field configurations within physiological safety limits—but small-bore preclinical performance cannot be linearly extrapolated to human-scale systems, and systematic data from human-scale prototypes operating in realistic tissue conditions remains the field’s most urgent experimental need. The biological validity gap is the hardest to close; most quantitative benchmarks rest on murine xenograft models, whose nanoparticle accumulation kinetics and vascular architecture differ substantially from human disease. Moving to patient-derived organoids, large-animal studies, and co-clinical trial designs is a methodological shift the field must now make.

The most realistic near-term path concentrates on indications where MPI’s advantages are strongest, and barriers are lowest: acute neurological care, where real-time perfusion mapping and simultaneous haemorrhage characterization address an unmet need no single existing modality covers, and quantitative cell tracking in regenerative medicine and immunotherapy, where depth-independent longitudinal quantification over weeks to months has no adequate alternative. MPI-guided theranostics and functional neuroimaging are the most compelling long-term directions, but require simultaneous progress across all three gaps before clinical translation is realistic.

The field is not waiting for a scientific breakthrough: the physics is sound, and the performance ceiling is high. It is waiting for the alignment of tracer chemistry, scanner engineering, and clinical study design around shared biological targets. That alignment, integrated, indication-driven, and tested in progressively realistic models, is both achievable and overdue, and the first clinical wins will come exactly where MPI offers something other modalities cannot provide at all.

## Figures and Tables

**Table 1:** Comparative analysis of various top-down and bottom-up synthesis methods for magnetic nanoparticles, highlighting fundamental principles, operational advantages, and inherent limitations.

Method	Basic Principle	Advantages	Disadvantages
Mechanical Milling	High-energy impact and shear force fracture bulk materials into nanoscale powders.	Well-suited for large-scale production; low operational cost; applicable to many metals.	Risk of product contamination; high equipment wear; heat generation can cause unwanted phase changes.
Laser Ablation (LASiS)	Laser irradiation of a solid target submerged in liquid, converting bulk material into a colloidal dispersion.	High chemical purity; no surfactants needed; highly tunable size and morphology.	Low productivity; high energy consumption; requires expensive, sophisticated laser systems; moderate polydispersity due to non-equilibrium particle formation.
Physical Vapour Deposition (PVD)	Converting a solid source to vapour and condensing it onto a substrate in a vacuum.	Produces high-purity products; uniform size distribution; precise control over crystallinity.	Requires specialized vacuum hardware; high capital and operational costs; complex setup; potential size variability depending on growth and collection conditions.
Co-precipitation	Alkaline precipitation of metal salts (e.g., Fe^2+^ and Fe^3+^) in aqueous solutions at moderate temperatures.	Inexpensive reagents; highly scalable; water-compatible; clinically established.	Broad size distribution; low crystallinity; sensitive to pH/temperature; prone to oxidation.
Thermal Decomposition	High-temperature breakdown of organometallic precursors in organic solvents with surfactants.	Gold standard for monodispersity; excellent control over size and shape; high crystallinity.	Uses toxic organic solvents; requires inert atmospheres; hard to scale; particles are initially hydrophobic.
Hydrothermal/Solvothermal	Chemical reactions in sealed autoclaves under high temperature and pressure over long durations.	High crystallinity and narrow size distribution; near-thermodynamic growth control; aqueous compatible.	Requires high-pressure equipment; long reaction times; batch-to-batch reproducibility issues.
Sol–Gel	Conversion of molecular precursors (sol) into a solid network (gel) via hydrolysis and condensation.	Excellent control over composition and surface chemistry; simple setup for complex shapes.	Prolonged reaction times; potential for byproduct contamination; often results in aggregated networks.
Green Synthesis	Using microorganisms or plant extracts to biologically reduce and stabilize metal precursors.	Environmentally friendly; high inherent biocompatibility; minimal post-synthetic modification needed.	High polydispersity (uneven sizes); lack of morphological uniformity; difficult to standardize.

## Data Availability

No new data were created or analyzed in this study.

## Abbreviations

The following abbreviations are used in this manuscript:

AMF	Alternating Magnetic Field
BBB	Blood–Brain Barrier
CA	Citric Acid
CAR-T	Chimeric Antigen Receptor T cells
CIONs	Cubic Iron Oxide Nanoparticles
CMD	Carboxymethyl Dextran
CNR	Contrast-to-Noise Ratio
CT	Computed Tomography
DMSA	Dimercaptosuccinic Acid
EPR	Enhanced Permeability and Retention
EVs	Extracellular Vesicles
FDA	Food and Drug Administration
Fe	Iron (elemental symbol)
FFL	Field-Free Line
FFP	Field-Free Point
FFR	Field-Free Region
FOV	Field of View
FWHM	Full Width at Half Maximum
IgG	Immunoglobulin G
LASiS	Laser Ablation Synthesis in Solution
LSI	Linear and Shift-Invariant
MNPs	Magnetic Nanoparticles
MPI	Magnetic Particle Imaging
MPS	Magnetic Particle Spectroscopy
Ms	Saturation Magnetization
NPs	Nanoparticles
OA	Oleic Acid
OAm	Oleylamine
PEG	Polyethylene Glycol
PEGylation	Polyethylene Glycol conjugation
PET	Positron Emission Tomography
PLGA	Poly(lactic-co-glycolic acid)
PMAO	Poly(maleic anhydride-alt-1-octadecene)
PNS	Peripheral Nerve Stimulation
PSF	Point Spread Function
PVA	Polyvinyl Alcohol
PVD	Physical Vapour Deposition
PVP	Polyvinylpyrrolidone
RES	Reticuloendothelial System
ROS	Reactive Oxygen Species
SE-MPI	Subtraction-Enhanced MPI
SM	System Matrix
SNR	Signal-to-Noise Ratio
SPIONs	Superparamagnetic Iron Oxide Nanoparticles
TME	Tumour Microenvironment
US	Ultrasound
